# Immune Dysfunction in Uremia 2020

**DOI:** 10.3390/toxins12070439

**Published:** 2020-07-05

**Authors:** Gerald Cohen

**Affiliations:** Department of Nephrology and Dialysis, Medical University of Vienna, Vienna A-1090, Austria; gerald.cohen@meduniwien.ac.at

**Keywords:** cardiovascular disease, infections, oxidative stress, inflammation, immune cells, autophagy, uremic toxins, renin-angiotensin- system, erythropoietin, vitamin D

## Abstract

Cardiovascular disease and infections are major causes for the high incidence of morbidity and mortality of patients with chronic kidney disease. Both complications are directly or indirectly associated with disturbed functions or altered apoptotic rates of polymorphonuclear leukocytes, monocytes, lymphocytes, and dendritic cells. Normal responses of immune cells can be reduced, leading to infectious diseases or pre-activated/primed, giving rise to inflammation and subsequently to cardiovascular disease. This review summarizes the impact of kidney dysfunction on the immune system. Renal failure results in disturbed renal metabolic activities with reduced renin, erythropoietin, and vitamin D production, which adversely affects the immune system. Decreased kidney function also leads to reduced glomerular filtration and the retention of uremic toxins. A large number of uremic toxins with detrimental effects on immune cells have been identified. Besides small water-soluble and protein-bound compounds originating from the intestinal microbiome, several molecules in the middle molecular range, e.g., immunoglobulin light chains, retinol-binding protein, the neuropeptides Met-enkephalin and neuropeptide Y, endothelin-1, and the adipokines leptin and resistin, adversely affect immune cells. Posttranslational modifications such as carbamoylation, advanced glycation products, and oxidative modifications contribute to uremic toxicity. Furthermore, high-density lipoprotein from uremic patients has an altered protein profile and thereby loses its anti-inflammatory properties.

## 1. Cardiovascular Disease and Infections as the Main Causes of Death in Uremia

Uremia literally means “urea in the blood” and is characteristic for chronic kidney disease (CKD) and end-stage renal disease (ESRD), but may also occur as a consequence of acute kidney injury [1]. CKD is one of the most severe health problems worldwide, leading to high economic costs to the health system [2,3]. CKD is defined by current international guidelines as reduced kidney function characterized by a glomerular filtration rate (GFR) of less than 60 mL/min per 1.73 m2 or markers of kidney damage, or both, of at least 3 months duration [4]. In CKD patients, the health-associated quality of life gradually declines with disease progression. In 2016, an estimated global incidence of between 11% and 13%, with the majority CKD stage 3, was reported [3]. The global all-age mortality rate from CKD increased 41.5% between 1990 and 2017 [5]. The permanently decreased glomerular filtration rate and proteinuria in CKD patients are associated with an increased risk of morbidity and mortality, mainly caused by cardiovascular disease (CVD) and infections [6,7,8].

Besides the risk of death due to vascular diseases and infections, neoplastic diseases contribute to the increased mortality of CKD patients [9]. The manifestation of cancer is a major comorbidity factor leading to the establishment of “onconephrology” as a new specialty in nephrology [10]. Among the hematopoietic tumors associated with CKD, multiple myeloma, and non-Hodgkin lymphoma, diseases related to alterations in the immune system have the highest incidence [11]. However, a Canadian study showed that there is an inverse association between the estimated glomerular filtration rate (eGFR) and the individual causes of death [12]. Whereas below an eGFR of 60 mL/min per 1.73 m^2^ the most common cause of death was CVD, cancer was the most common reason of death above an eGFR of 60 mL/min per 1.73 m^2^.

The morbidity and mortality profiles of CKD patients are remarkably similar to those of the geriatric population, especially with regard to alterations in their vascular and immune systems [13]. In uremic patients, immune dysfunction and low-grade inflammation leading to increased susceptibility for both cardiovascular and infectious diseases have parallels with the general aging process [14].

CVD can be observed in all stages of CKD [15]. However, the occurrence of cardiac events markedly rises during the progression of kidney damage and reaches its maximum at ESRD [6,15]. At an estimated GFR < 45 mL/min/1.73 m^2^, the risk of cardiovascular mortality increases distinctly with decreasing GFR [16].

The majority of hemodialysis (HD) patients have CVD and their mortality rate caused by CVD is 20 times higher than in the general population [17]. Furthermore, dialysis patients have increased annual mortality rates caused by sepsis, also after stratification for age, race, and diabetes mellitus [18]. In general, preexisting medical conditions affect the clinical course of sepsis. Of note, CKD is associated with higher 90-day mortality than any other chronic medical conditions in patients with sepsis [19].

Infection is the second main cause of death in patients with reduced renal function. The incidence of mortality varies between 12% and 22% [20]. Patients with CKD not undergoing dialysis treatment have a higher risk of bloodstream infection, which is associated with an estimated GFR less than 30 mL/min/1.73 m^2^ [21]. Another cause for infections is an insufficient response to vaccinations as a consequence of a deficient T-lymphocyte-dependent immune response [22].

On one hand, kidney failure affects the general immunity, resulting in intestinal barrier dysfunction, systemic inflammation, and immunodeficiency; conversely, kidneys may be targets of pathogenic immune responses against renal autoantigens and of local effects of systemic autoimmunity [23].

**Figure 1:**
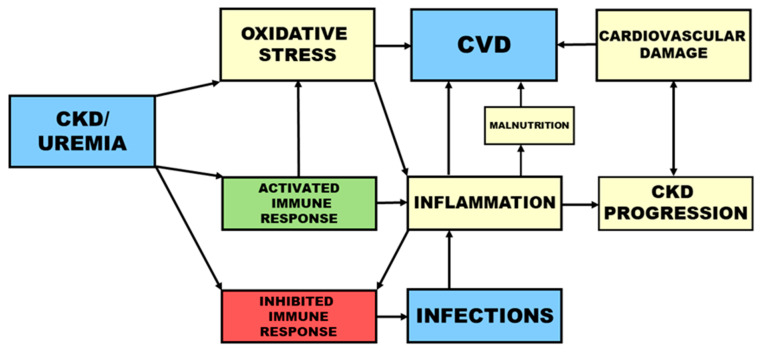
Immune dysfunction and risk factors in chronic kidney disease.

In uremia, the increased risk for both cardiovascular events and infections is associated with a disturbed immune response. Whereas an attenuated immune defense contributes to the high occurrence of infections, pre-activation and priming of immune cells leads to inflammation and consequently to CVD (Figure 1).

## 2. Immune Cells in Uremia

### 2.1. Polymorphonuclear Leukocytes (PMNLs)

Polymorphonuclear leukocytes (PMNLs) are crucial elements of the non-specific cellular immune defense and participate in the primary immune reaction. They have a short circulating half-life of 4 to 18 h [24]. PMNLs, named after their lobulated nuclei, contain multiple granules in their cytoplasm. Therefore, they are also commonly referred to as granulocytes. According to the staining behaviors of their granules, three groups are distinguished: eosinophilic, basophilic, and neutrophilic granulocytes. Neutrophils make up the largest group of white blood cells and play an essential role in the defense against bacterial and fungal infections. The recruitment of neutrophils and their function in health and under inflammatory conditions has been previously reviewed [25].

Disturbed functions of PMNLs lead to an enhanced risk of bacterial infections and represent a main cause for the increased risk of morbidity and mortality among CKD patients [26]. After chemotactic movement to the source of infection, PMNLs take up the invading microorganisms by phagocytosis and kill them with toxic oxygen radicals formed during the oxidative burst and proteolytic enzymes intracellularly released from granula. Disturbances of any of those crucial PMNL functions increase the risk for bacterial infections. The proneness of CKD patients to infections resulting from decreased phagocytosis is caused by factors such as uremic toxins, iron overload, anemia of renal disease, and dialyzer bioincompatibility [27].

Neutrophils from HD patients show significantly elevated levels of reactive oxygen species (ROS) production, degranulation, and basal neutrophil extracellular trap (NET) formation, indicating spontaneous activation [28]. Furthermore, similar to PMNLs from patients with acute infections, PMNLs from HD patients show the expression pattern CD16(bright)/CD62L(dim), whereas cells from healthy subjects were normal CD16(bright)/CD62L(bright) [28].

The coordinated elimination of activated PMNLs is essential for the resolution of inflammation [29]. Increased apoptosis leads to a reduced immune response, whereas delayed apoptosis of PMNLs or compromised clearance of apoptotic PMNLs by macrophages causes inflammation [30].

A rise in the concentration of intracellular calcium ([Ca^2+^]_i_) is a key second messenger in PMNLs [31,32] and modulates essential PMNL functions and apoptotic cell death [33,34,35]. PMNLs from HD patients have an augmented basal [Ca^2+^]_i_ [36], which is associated with reduced reactivity upon stimulation [37]. Besides the regulation of pro-inflammatory responses, Ca^2+^ -signaling plays an important role in cytokine secretion and the formation of NETs [38].

#### 2.1.1. Neutrophil Extracellular Traps

Beside microbial uptake and the secretion of antimicrobials, neutrophils release NETs to eliminate invading microbes including bacteria, fungi, and parasites. NETs are networks of extracellular fibers, mainly composed of neutrophil DNA and granular antimicrobial proteins. The process of NET generation, called NETosis, is a specific type of cell death and is different from apoptosis and necrosis. Neutrophils form NETs upon contact with various bacteria, fungi, and activated platelets as well as under the influence of inflammatory stimuli. Pathogens trapped in NETs are killed by oxidative and non-oxidative mechanisms [39]. NETs also represent a physical barrier and a framework enhancing antimicrobial synergy and reducing harm to host tissues [40]. Beside this suicidal NETosis, a different type of NETosis—termed vital NETosis—has been described [41]. DNA- containing vesicles are released without the disruption of the plasma membrane, leaving the PMNLs viable and still able to phagocytose and migrate.

Increased circulating nucleosome levels in HD patients are closely associated with concentrations of myeloperoxidase (MPO), a lysosomal protein stored in neutrophil granules, indicating elevated levels of NETs in vivo [42]. Patients with the highest NETs levels had a significantly increased all-cause and cardiovascular mortality, even after adjusting traditional risk factors [42]. Associations between elevated cell-free DNA levels in dialyzed patients and the process of NETosis have been recently reviewed [43]. NETs are involved in the development of comorbidities in HD patients, e.g., by stimulation of thrombosis and endothelial damage. Therefore, NETosis may be considered as a link between neutrophil activation during the HD procedure and comorbidities in dialyzed patients [43].

The DNA-complexed granular proteins and other proteins released by neutrophils during NETosis may lead to autoimmunity syndromes such as systemic lupus erythematosus, small-vessel vasculitis, or autoimmune diseases associated with the formation of autoantibodies against chromatin and neutrophil components [44].

#### 2.1.2. PMNL Priming

Priming of immune cells is the amplification of a reaction to a stimulus caused by preceding contact with a priming agent, whereas the elevation of basal activation states is called pre-activation [45]. The activation status of neutrophils can cycle from basal through primed to fully activated [46]. The ability of primed cells to revert to a basal state gives them functional flexibility in the modulation of PMNL activities at sites of inflammation [46]. In the presence of normal plasma, the oxidative burst of uremic PMNL can return from a primed to a non-primed state, indicating the presence of priming factors in uremic plasma [47]. Furthermore, priming of PMNLs attenuates their constitutive apoptosis [48].

Whereas controlled neutrophil priming has a beneficial role in host defense, excessive neutrophil priming in inflammatory diseases has harmful effects [49]. Primed and activated cells show significantly increased bactericidal capacity such as augmented respiratory burst activity and degranulation [46]. Inappropriate PMNL priming is a central mediator of low-grade inflammation and oxidative stress in CKD patients [50]. Furthermore, primed neutrophils can lead to increased NETs formation under uremic conditions and are associated with endothelial dysfunction [42].

### 2.2. Monocytes

Monocytes are bone marrow derived cells that circulate in the blood for 1-3 days before differentiating into tissue macrophages or dendritic cells. In a review in this special issue, Girndt et al. [51] focuses on the differences of uremic monocytes in the expression of surface molecules, the production of cytokines and mediators, and in their function as compared to monocytes of healthy persons. Based on their CD14/CD16 expression profile, three distinct populations of monocytes can be distinguished: Mo1 (“classical monocytes” expressing CD14 only), Mo2 (“intermediate monocytes”, CD14++/CD16+), and Mo3 (“non-classical monocytes”, CD14+/CD16++). The Mo2 population has pronounced pro-inflammatory properties. Their proportion is an important predictor of mortality risk in HD patients [52]. The contribution of monocytes to the dysregulated immune response in uremia can be based on altered features of the individual cells or on a change in the relative numbers of the three different populations.

### 2.3. Dendritic Cells

The functions of dendritic cells, which link innate and adaptive immunity by presenting antigens, are disturbed in HD patients. The terminal differentiation of monocyte-derived dendritic cells is compromised in advanced CKD and independent of HD treatment [53]. An antigen-presenting dendritic cell dysfunction, beside a T-cell defect, can cause a diminished response to vaccination in ESRD patients [54]. Dendritic cells are also present in the kidney and contribute to the progression of renal failure by binding to glomerular antigens [55]. Their presentation to infiltrating T cells leads to the production of pro-inflammatory cytokines and activation of additional immune effector cells, which are key components of the tubulointerstitial mononuclear infiltrate that is typical for progressive renal disease [56].

### 2.4. Lymphocytes

Similar to innate immunity, acquired immune deficiency contributes to the high morbidity and mortality of ESRD patients [57]. In a review in this special issue, Betjes et al. [58] summarize the current data about uremia-associated ageing of the thymus and its role in dysfunctional adaptive immune responses. Of note, kidney transplantation is not able to restore the compromised thymus function. The main characteristic of immunological ageing is naïve T cell lymphopenia combined with the expansion of highly differentiated memory T cells, which have a pro-inflammatory phenotype destabilizing atherosclerotic plaques and contributing to an inflammatory state. In ESRD, the decreased number of naïve B cells in the entire B cell population contributes to the compromised adaptive immune response [59].

## 3. Oxidative Stress and Inflammation

Various clinical models of CKD, including diabetic nephropathy, IgA nephropathy, polycystic kidney disease, and the cardio-renal syndrome, are associated with oxidative stress [60]. Oxidative stress increases in parallel with the development of CKD [61]. Markers of oxidative stress predict the survival of HD patients [62]. Moreover, antioxidant systems are compromised in CKD patients and deteriorate gradually with the degree of renal failure [63]. Oxidative stress evokes inflammation via the formation of pro-inflammatory oxidized lipids or advanced oxidation protein products (AOPPs), while stimulation of NFκB in the pro-oxidant environment promotes the expression of pro-inflammatory cytokines and recruitment of pro-inflammatory cells [60]. Whereas oxidative stress and inflammation are normally protective against infections and physiological responses to harmful stimuli, several deleterious effects are induced if they become uncontrolled, maladaptive, and persistent in ESRD [64]. Uremic toxins such as methylglyoxal (MGO) enhance the oxidative burst of PMNLs [65].

Nuclear factor erythroid 2-related factor 2 (Nrf2), a basic leucine zipper protein, regulates the expression of antioxidant proteins that protect against oxidative damage. An integrative biology approach based on single-nucleotide polymorphisms (SNPs) generated a molecular map of CKD. Nrf2-mediated oxidative stress represented a link between inflammation and metabolism-related pathways associated with CKD [66]. Considering that genetic features play an important role in the development and prognosis of CKD, Jerotic et al. [67] investigated the association between the polymorphism in Nrf2, superoxide dismutase, and glutathione peroxidase and showed that polymorphisms in these genes are associated with the development of ESRD and can predict survival.

Inflammation can be caused by oxidative stress and infections (Figure 1) and by factors related to HD treatment such as biocompatibility and dialysate quality [68]. Bacterial DNA in the dialysate leads to increased oxidative stress and higher serum levels of hsCRP and IL-6. Furthermore, a lower concentration of pre-dialysis plasma bicarbonate and the resulting decreased intracellular pH value in HD patients contribute to oxidative stress [69].

Chronic renal and vascular oxidative stress in association with an increased inflammatory burden contribute to the development and progression of diabetic complications including CVD, atherosclerosis, and diabetic kidney disease (DKD) [70]. Worldwide, diabetes mellitus is the most common cause of CKD [71,72].

Besides CVD, inflammation is also associated with protein-energy wasting and malnutrition, inflammation, and atherosclerosis syndrome [73]. Malnutrition itself is an essential risk factor for the development of CVD [74] (Figure 1). The development of CVD among CKD patients is associated with metabolic changes, i.a. in lipid profile and serum concentrations of CRP and homocysteine [75].

## 4. Toll-Like Receptors and Inflammasomes

Toll-like receptors (TLRs) are pattern recognition receptors that, together with inflammasomes, detect and react to highly conserved motifs on pathogens (pathogen-associated molecular patterns; PAMPs) and to substances released upon cell damage or stress (damage-associated molecular patterns; DAMPs) [76]. In CKD, TLRs regulate inflammatory and tissue-repair responses to infection and injury. Aberrant stimulation of pattern recognition receptors may result in immunodeficiency, septic shock, and autoimmunity [77]. Upon recognition of pathogens via TLRs, monocytes and PMNL induce cellular activation and secretion of inflammatory cytokines. In HD patients, the expression of TLR2 and TLR4 on monocytes and TLR4 on PMNLs is increased, leading to an elevated production of cytokines after TLR4 stimulation by endotoxin [78]. However, Baj et al. found that despite chronic inflammation in ESRD patients, the expression of TLR4 and TLR9 on PMNLs was not significantly different from controls [79]. In sterile inflammatory and immune-mediated kidney diseases, TLRs can increase and self-perpetuate tissue injury [76]. Sterile inflammation mediated by DAMPs is a physiological response of the immune system to tissue injury in the absence of infection. Most innate immune pathways that detect infection are involved in sterile inflammation [80]. Triggers of sterile inflammation are nuclear proteins or mitochondrial components such as formylated proteins, DNA, and ATP.

Inflammasomes are multiprotein complexes involved in innate immunity and regulate caspase-dependent inflammation and cell death [81]. After detection of pathogens or danger signals in host cells, they are assembled by pattern recognition receptors. Dysregulated inflammasome activity is associated with hereditary and acquired inflammatory disorders [82]. By affecting inflammation, pyroptosis, apoptosis, and fibrosis, the NLRP3 inflammasome contributes to a variety of acute and chronic microbial and non-microbial kidney diseases [81].

Hypervolemia (fluid overload) correlates with cardiovascular risk factors in patients with CKD [83] and is a main cause of hypertension, heart failure, and mortality in HD patients [84]. Inflammation is usually higher in hypervolemic HD patients compared to normovolemic patients. The combination of hypervolemia and inflammation, both independent risk factors for mortality, can lead to a cumulative risk profile [85]. Ulrich et al. [86] assessed the hypervolemic activation of peripheral blood mononuclear cells of HD patients with special emphasis on the NLRP3 inflammasome response. They found that the NLRP3 inflammasome is not activated by hypervolemia, suggesting that endotoxemia is not a main driver for inflammation in hypervolemic HD patients.

## 5. Autophagy

Autophagy is a controlled mechanism of cells, leading to a systematic degradation and recycling of defective and unnecessary cell components [87]. It has both anti- and pro-inflammatory effects. After autophagy inhibition, NETs were significantly more abundant, indicating a protective role of autophagy in excessive NET formation [88]. Autophagy supports productive and inhibits over-exuberant inflammatory responses to avoid excessive tissue damage and ensure adequate responses [89].

Autophagy has a role in both innate and adaptive immunity and can interfere with bacterial pathogens at several steps of invasion and directly eliminate intracellular microorganisms. In innate immunity, autophagy reduces inflammation by suppressing the activation of inflammasomes via the removal of protein aggregates, degradation of damaged mitochondria, and elimination of inflammasome components [90,91]. Autophagy affects the maturation, homeostasis, function, and polarization of T cells. Furthermore, autophagy controls autoimmune responses by modulating innate immune functions and lymphocyte homeostasis [92]. Autophagy is also important in innate recognition of viral pathogens and IFN-α production in plasmacytoid dendritic cells and the transport of cytosolic viral replication intermediates into the lysosome [93].

Autophagy plays a prominent role in renal physiology and homeostasis [94] and is renoprotective in epithelial renal cells and podocytes in various models of acute kidney injury, glomerular disease, and ageing [95]. Inflammation and mitochondrial dysfunction in kidney diseases can lead to disturbed cellular recycling by affecting autophagy activation and inhibition. Autophagy is a general response to oxidative stress in cells and tissues [96].

In CKD, the activation of leukocyte autophagy is impaired and cannot be restored by HD treatment [97]. On the other hand, autophagy is increased early after sepsis and protects organs from pathogen by regulating macrophage, dendritic cells, B cells, CD4+, and CD8+ T cells functions [98].

## 6. Complement System

The complement system is an important part of the immune defense and a link between innate and adaptive immunity. It boosts the clearance of microorganisms and damaged cells by phagocytes and antibodies. Uncontrolled activation of the complement pathways can cause indirect immune-mediated renal disease [99]. Complement over-activation is involved in the pathogenesis of C3 glomerulopathy and atypical hemolytic uremic syndrome (aHUS) [100]. Neutrophils may adhere to complement-activated endothelial cells, form aggregates with platelets on endothelial cells, and thereby contribute to the manifestation of aHUS [101]. In both C3 glomerulopathy and aHUS, defective regulatory factors of the alternative pathway are observed [102]. The complement system is also involved in the development of acute kidney injury (AKI) [103] as well as increased risk for the development of CKD after the occurrence of AKI (AKI-to-CKD transition) [104]. Furthermore, complement activation can also contribute to systemic inflammation, leading to remote organ injury [103].

## 7. Metabolic Functions of the Kidney

Besides elimination of toxic uremic retention solutes, the kidney has several crucial metabolic functions related to the immune system, such as the production of renin, calcitriol, and erythropoietin (EPO).

### 7.1. Renin-Angiotensin-System (RAS)

The kidney secretes renin when blood pressure is low and thereby stimulates the production of angiotensin. The renin-angiotensin system (RAS) regulates blood pressure by modulating the vascular tone and is involved in the pathogenesis of inflammation and CKD progression. RAS activation leads to high levels of angiotensin II and aldosterone and contributes to further worsening of kidney injury via TGF-β and promotes CVD through sodium retention and vasoconstriction, which lead to hypertension (reviewed in [105]). Besides angiotensin II, several other RAS enzymes, such as angiotensin-converting enzyme 2 (ACE2), affect immune homeostasis. Via Ang1–7 generation, ACE2 reduces AngII levels and thereby blunts inflammatory responses caused by AngII. The immunological effects of the RAS are thoroughly described in a recent review [106].

Monocytes of HD and not-dialyzed CKD stage 3–5 patients have an increased ACE and a reduced ACE2 expression and therefore may accelerate the development of atherosclerosis [107]. The increased ACE expression on monocytes of dialysis patients with CVD provides a link between monocytes and the activated RAS [108]. Of note, circulating miR-421 targeting leukocytic ACE2 are increased in CKD patients [109].

T and natural killer cells express all RAS elements, implying that they can bring angiotensin II to sites of inflammation [110]. Angiotensin II stimulates PMNL oxidative burst and increases [Ca^2+^]_i_ [111]. However, RAS stimulation in hematopoietic cells may have other immunologic effects than RAS activation in the kidney and vasculature. Activation of type 1 angiotensin (AT1) receptors in T lymphocytes and myeloid cells reduces the polarization toward pro-inflammatory phenotypes and thereby protects the kidney from hypertensive damage and fibrosis [106].

### 7.2. Erythropoietin and Iron

Decreased systemic and local oxygen tension causes the production of EPO by the kidney. EPO triggers the formation of red blood cells in the bone marrow and is under the transcriptional control of the hypoxia-inducible factor-2α, which mediates a reduced degradation of EPO by proteasomes in hypoxia. During hypoxic stress, the levels of EPO are elevated 1000-fold. Its naturally short half-life of 5 to 8 h is controlled by glycosylation [112].

The decreased serum level of EPO is a main cause of CKD-associated anemia. In addition to the reduced EPO production by the diseased kidney, the presence of erythropoiesis inhibitory substances in uremic sera is supported by the observation that sera from dialysis patients are able to inhibit the growth of the human leukemic cell line UT-7, whereas sera from healthy controls showed no effect [113].

Ever since clinical trials in 1987 proved the efficacy of recombinant human EPO (rhEPO) for the treatment of anemia in CKD, EPO therapy has been established [114]. However, a pronounced variability in the response to rhEPO has been noticed. Absolute and functional iron deficiency as well as chronic inflammation, which affects erythropoiesis via pro-inflammatory cytokines—for example, IL-1, TNFα, and interferon-γ [115]—can lead to rhEPO resistance. Iron is required for the formation of hemoglobin and is an essential nutrient. Intravenous iron therapy preserves iron stores and lowers the need for EPO in HD patients. However, iron therapy affects leukocyte functions and cytokine production, stimulates oxidative stress, and promotes bacterial growth. Furthermore, iron therapy of CKD patients adversely affects essential functions of phagocytes and T and B lymphocytes [116]. However, in a recent nationwide cohort-based case-crossover study in Taiwan, intravenous iron supplementation did not increase the short-term infection risk among HD patients [117].

Besides its erythropoietic function, EPO possesses immunomodulatory properties. The positive effect of rhEPO therapy on the immune defense is not only a consequence of anemia correction, but also has a direct effect on immunological responses [118]. The EPO-receptor is expressed on PMNLs [119], lymphocytes, and monocytes [120]. EPO up-regulates TLR4 in differentiating dendritic cells, making them more susceptible to stimulation by the TLR4 ligand lipopolysaccharide [121]. In kidney transplant patients, the improved outcomes associated with the correction of anemia with EPO is erythropoiesis-independent and caused by inhibition of T-cell immunity via the EPO-receptor on T cells [122].

#### Hepcidin

Hepcidin, a 25 amino acid peptide synthesized by the liver, is a main regulator of iron homeostasis. By binding to the cellular iron exporter ferroportin, it controls the distribution of iron in the body. Inflammation increases the hepcidin level, resulting in serum hypoferremia and tissue hyperferritinemia [123]. In CKD, hepcidin may be considered as a link between inflammation and anemia in CKD [124]. The excessive production of hepcidin causes relative deficiency of iron during inflammatory states, resulting in functional iron deficiency in anemia of inflammation [125]. Under those conditions, hepcidin levels may increase up to 100-fold [126]. However, elevated hepcidin levels in CKD can be attenuated by EPO therapy [127]. In patients with CKD (3b-5) from a prospective study in Korea (2011-2016), serum hepcidin concentrations were inversely associated with eGFR [128]. In contrast, markers of inflammation and iron status were positively associated with serum hepcidin level, irrespective of CKD stage [128].

### 7.3. Vitamin D, Fibroblast Growth Factor 23, and Parathyroid Hormone

The active vitamin D metabolite calcitriol (1,25-dihydroxy-vitamin D, 1,25-dihydroxy-cholecalciferol) is produced in the kidney and in extra-renal tissues such as activated monocytes/macrophages [129] and endothelial cells. In patients with CKD, hyperphosphatemia, the main inhibitory signal, and parathyroid hormone (PTH), the main stimulatory signal for 1alpha-hydroxylase, the rate-limiting enzyme of calcitriol synthesis, are modified and contribute to the reduced calcitriol production resulting in calcitriol deficiency [130]. The production of vitamin D may be inhibited by uremic retention solutes [131]. Furthermore, calcitriol is less effective in uremia.

Besides its influence on the regulation of calcium, phosphate, and parathyroid hormone, vitamin D regulates cell differentiation/proliferation and the immune system [132]. The modulation of the immune system, controlling of inflammatory responses, and suppression of the RAS can delay the development of CVD [133]. The suppression of the local RAS in mice via macrophage vitamin D receptor signaling can inhibit atherosclerosis [134]. In an in vitro study with B and T lymphocytes from healthy subjects, uremic serum increased the intracellular expression of IL-6 and TLR9. This effect could be reduced by 25-hydroxy- or 1,25-dihydroxy-vitamin D, showing that cholecalciferol repletion has an anti-inflammatory effect and improves vitamin D intracellular regulatory enzymes in lymphocytes from dialysis patients [135]. However, no convincing evidence for the use of vitamin D therapy in improving cardiovascular outcomes could be shown in several meta-analyses and randomized clinical trials [136].

With decreasing kidney function, rising serum phosphate levels induce the production of the bone-derived phosphaturic hormone fibroblast growth factor 23 (FGF23), which is crucial for the regulation of vitamin D and phosphate homeostasis [137]. Serum FGF23 increases early in the progression of CKD and can reach extremely high levels in HD patients. FGF23 concentrations rise 1000-fold above normal values in order to keep the phosphate levels within the normal range and are associated with disease progression [137]. In ESRD, FGF23 fails to sustain phosphate homeostasis and hyperphosphatemia and increased FGF23 levels stimulate the development of hypertension, vascular calcification, and left ventricular hypertrophy [138]. In CKD patients, there is a dose-dependent association between circulating FGF23 levels and an increased risk of premature mortality [139,140].

In both HD patients and kidney transplant recipients, FGF23 is a strong predictor of all-cause and cardiovascular mortality [141]. However, in Japanese HD patients, no association between FGF23 levels and with parameters of cardiac dysfunction, atherosclerosis, infection, and systemic inflammation could be found, not supporting the hypothesis that high FGF23 in dialysis patients is the cause of cardiac dysfunction, atherosclerosis, infection, or systemic inflammation [142].

The transmembrane protein klotho is a co-receptor for FGF-23, with a clinical impact on mineral metabolism. Low klotho levels are associated with cardiovascular events in HD patients, independently from factors associated with mineral-bone disease [143]. In CKD–mineral and bone disorder, dysregulated FGF23, klotho, vitamin D, and PTH cause progressive hyperphosphatemia, hypercalcemia, and hyperparathyroidism and contribute to the increased cardiovascular risk in patients with renal failure [144].

Parathyroid hormone levels are increased in CKD patients. In a recent review, Duque et al. [145] describe the significance of parathyroid hormone as a uremic toxin contributing to the manifestations of the uremic syndrome. In CKD, secondary hyperparathyroidism leads to elevated FGF-23 serum levels, decreased calcitriol production in the kidney, reduced intestinal calcium absorption, and hyperphosphatemia. Chronic excess of parathyroid hormone in uremia also disturbs PMNL functions through continuous elevation of their [Ca^2+^]_i_ [37]. Parathyroidectomy lowers but does not lead to a normal range of PMNL [Ca^2+^]_i_ of CKD patients [146]. This suggests that other factors such as uremic retention solutes affect [Ca^2+^]_i_ and PMNL functions. Elevated parathyroid hormone concentrations in CKD also affect functions of B cells [147] and T-lymphocyte functions and thereby contribute to altered cellular immunity [148].

## 8. Uremic Toxins

The development of the uremic syndrome is initiated by the retention of substances normally filtered by the healthy kidneys. Retention solutes that exert a detrimental effect on biologic functions are called uremic toxins [149].

In 2003, the European Uremic Toxin Work Group (EUTox) compiled a comprehensive list of 90 uremic retention solutes known at that time [150]. Uremic retention solutes have been categorized according to their physicochemical properties into small water-soluble molecules, middle molecules, and protein-bound molecules [150]. In 2012, this classification of normal and pathologic levels of uremic retention solutes was extended [151]. An updated database of “uremic toxins and solutes that accumulate in the plasma during the later stages of CKD” can be found at the homepage of EUTox (https://www.uremic-toxins.org/).

Because uremic toxins that lead to vascular complications are also produced in the intestine [152], Meijers et al. suggested another classification considering the source of metabolites, originating, therefore, from the diet, the mammalian, or the microbial metabolism [153].

In a review in this special issue, Glorieux et al. [154] describe the pathophysiological effects of intestinally generated end-products of the bacterial metabolism such as *p*-cresol, trimethylamine, and hydrogen sulfide (H_2_S). The role of the intestinal microbiota in the accumulation of sulfur compounds has been recently reviewed by Perna et al. [155]. The human immune system is crucial in preserving homeostasis with the resident microbiota, whereas in return, resident microbial populations affect the human immune defense [156]. Under inflammatory conditions in CKD, uremic toxins of bacterial origin disturb the intestinal barrier function. In the circulation, those uremic toxins stimulate immune cells. The abnormal gut microbiota in ESRD patients shapes a harmful metabolome, worsening clinical outcomes. Therefore, the intestinal microbiome represents a promising target to reduce uremic toxin levels [157,158].

Recently, mitochondria have been described as a source of and contributors to the production of uremic toxins [159]. The functioning of mitochondria can directly affect the synthesis of uremic toxins, such as products of oxidation or of peroxidation of cell components. Because uremic toxins can lead to damage of mitochondria, which gives rise to further uremic toxins, a positive feedback loop develops, leading to increased production of uremic toxins.

Uremic toxins that have inhibitory and/or pro-apoptotic effects on immune cells contribute to the increased risk of infections, whereas baseline activation, priming, and/or anti-apoptotic features lead to inflammation and as a consequence, to CVD. Examples of uremic toxins with known cardiovascular effects are the protein-bound solutes indoxylsulfate (IS) and the conjugates of p-cresol, p-cresyl sulfate (pCS), and p-cresyl glucuronide, the small water-soluble solutes guanidines, such as asymmetric dimethylarginine (ADMA) and symmetric dimethylarginine (SDMA), and the middle molecules beta-2-microglobulin, interleukin-6, TNF-alpha, and FGF-23 [160]. Recently, Vanholder et al. published a comprehensive update on the biochemical and clinical impact of organic uremic retention solutes [161].

Noteworthy, Rroji et al. found that uremic solute concentrations [blood, urea, nitrogen (BUN), uric acid, creatinine, ADMA and SDMA, beta2-microglobulin, and a large array of protein-bound solutes] in CKD patients with advanced age are not significantly associated with their calendar age [162].

### 8.1. Protein-Bound Solutes

In this special issue, Espi et al. [163] reviews the role of protein-bound uremic retention solutes in CKD-associated immune dysfunctions with a special focus on tryptophan catabolites such as indoxyl-sulfate (IS) and cresol derivatives such as pCS.

IS-mediated dysfunction of monocytes and T cells provokes endothelial damage in ESRD [164]. Pletnick et al. provided in vivo evidence that IS, pCS, and p-cresyl glucuronide have pro-inflammatory effects, contributing to vascular damage by stimulating a crosstalk between leukocytes and vessels [165]. pCS, the main in vivo metabolite of p-cresol, has a pro-inflammatory effect on unstimulated leukocytes by activating free radical production [166]. pCS induces the activation of macrophage, but interferes in antigen processing, resulting in a dysfunctional adaptive immune response in CKD patients [167].

The uremic retention solute para-hydroxy-hippuric acid reduces apoptosis of PMNLs from healthy subjects but has no effect on cells from hemodialysis patients, which may be desensitized by the uremic milieu [36]. Kynurenines, which belong to the group of indoles, are associated with oxidative stress, inflammation, and the occurrence of CVD in ESRD patients [168]. Under flow conditions, kynurenic acid initiates the stable arrest of leukocytes on the vascular endothelium. Therefore, it could be an important early mediator of leukocyte recruitment [169].

### 8.2. Small Water-Soluble Compounds

*In vitro*, the guanidino compounds SDMA, creatine, and guanidinobutyric acid (GBA) have a stimulatory effect on monocytes and granulocytes and may therefore contribute to the cardiovascular damage in CKD [170]. The post-translationally formed non-proteinogenic amino acids ADMA and SDMA are uremic toxins that inhibit the production of nitric oxide (NO) and are strong predictors of cardiovascular risk and mortality hemodialysis patients [171,172].

The purines uric acid, xanthine, and hypoxanthine suppress basal and calcitriol-induced CD14 expression of monocytes and are potentially relevant for impaired macrophage activation in renal failure [173]. The uremic toxin phenylacetic acid increases the activation of essential PMNL functions, reduces PMNL apoptosis, and may therefore account for the inflammatory state in uremic patients by priming PMNLs [174]. The uremic retention solutes dinucleoside polyphosphates (Np(n)N) stimulate the oxidative burst of PMNLs, monocytes, and lymphocytes and thereby account for the development of atherosclerosis in CKD [175].

The concentration of plasma MGO significantly increases with decreasing kidney function. MGO induces apoptosis of monocytes [176] and accelerates the spontaneous apoptosis and oxidative burst of PMNLs [177].

### 8.3. Middle Molecules

#### 8.3.1. Immunoglobulin Light Chains

Immunoglobulin light chains (IgLCs) are produced by B cells slightly in excess of Ig heavy chains in parallel to intact immunoglobulins [178]. As a result, they exist in their free, unbound form in the plasma of healthy people at low concentrations. Serum levels of free IgLCs are augmented either by reduced elimination, e.g., in patients with diminished kidney function, or as consequence of increased synthesis, e.g., in B-cell lymphoproliferative disorders such as multiple myeloma. In CKD patients, the concentrations of polyclonal free IgLCs increases gradually with CKD stage [179]. Whereas standard HD and HDF cannot normalize their serum concentration [180], extended HD with a protein-leaking dialyzer can remove large amounts of free IgLCs from the plasma of patients with myeloma and renal failure [181]. The novel medium cut-off (MCO) membranes are more efficient in removing IgLCs than classical high-flux membranes in hemodiafiltration and have a lower albumin loss [182].

IgLCs can interfere with several essential PMNL functions. Monomers and dimers of free polyclonal IgLCs isolated from HD and CAPD patients significantly inhibit PMNL chemotaxis in vitro [183]. IgLCs attenuate the stimulation of the PMNL glucose uptake, but increase its basal level. Since the uptake of glucose is a quantitative measurement of the activation state of phagocytic cells, these results suggest that free IgLCs are involved in the pre-activation of PMNLs and delay with the normal resolution of inflammation. In agreement with these findings, we previously showed that free IgLCs inhibit spontaneous apoptosis of PMNLs [184].

#### 8.3.2. Retinol-Binding Protein

The concentration of retinol-binding protein 4 (RBP4), the only specific vitamin A (retinol) transporter in blood, is increased in CKD [185]. In human umbilical vein endothelial cells, RBP4 induces apoptosis through receptor-mediated signaling [186] and inflammation by an NADPH oxidase- and nuclear factor kappa B-dependent and retinol-independent mechanism [187]. RBP isolated from the ultrafiltrate of patients with acute renal failure inhibits PMNL chemotaxis, oxidative burst, and apoptosis and as a result, contributes to a disturbed immune defense [188].

#### 8.3.3. Neuropeptides

Immune cells release opioid peptides and express the corresponding receptors. Therefore, opioids mediate the communication between immune and neuroendocrine systems. In vitro and in vivo studies have shown that opiate abuse impairs innate immunity and is responsible for increased susceptibility to bacterial infections [189]. In CKD, Met-enkephalin (Met-enk) levels are significantly increased in uremic serum and correlate with creatinine and urea concentrations [190]. Met-enk induces an up-regulation of CD11b and CD18 molecules on neutrophils in vitro [191]. Preliminary data from our laboratory suggest that Met-enk attenuates PMNL apoptosis and enhances PMNL chemotaxis in vitro [192]. We only observed these effects at concentrations higher than those reported in uremic serum. However, these effects could have in vivo relevance considering the autocrine action of Met-enk on PMNLs.

The levels of neuropeptide Y (NPY) are increased in CKD [193]. Plasma NPY concentrations predict incident CV complications in ESRD patients [194]. NPY, the most abundant peptide in the central and peripheral nervous system, acts as a signaling molecule at the interface of the immune system with the brain [195]. Human neutrophils express specific NPY receptors, which modulate essential neutrophil functions such as phagocytosis and oxidative burst [196]. NPY can be produced by immune cells upon stimulation and therefore regulate immune cell functions in an autocrine/paracrine manner [197].

#### 8.3.4. Endothelin-1

In CKD patients, especially those undergoing HD or CAPD treatment, plasma levels of endothelin-1 (ET-1), a potent coronary vasoconstrictor, are increased [198]. ET-1 is an important mediator for PMNL recruitment in adaptive inflammation via a TNFα and chemokine (CXCL1/CXCR2)-dependent mechanism [199]. ET-1 causes enhanced expression of leukocyte adhesion molecules and the synthesis of inflammatory mediators, leading to vascular dysfunction [200]. ET-1 promotes neutrophil adhesion to human coronary artery endothelial cells via ET(A) receptors [201]. PMNLs can produce ET-1 by proteolytic cleavage from human big endothelin (bET) [202]. PMNLs and macrophages express ET receptors. Therefore, considering the overproduction of ET-1 following endothelial dysfunction and inflammation, they may contribute to vascular dysfunctions by formation of an autocrine loop between ET-1 and the ET(A) receptor [203]. Furthermore, ET-1 enhances superoxide generation of human PMNLs stimulated by the chemotactic peptide N-formyl-methionyl-leucyl-phenylalanine [204].

#### 8.3.5. Adipokines

Adipose tissue has pleiotropic functions far beyond energy storage [205]. It plays a key role in cytokine, adipokine, and chemokine secretion as well as in the innate immune response [206]. As stated in the recent literature, adipokines have the highest association with CKD and ESRD [207]. Interactions of the adipose tissue with the kidney (adipo-renal axis) are important for normal kidney function and response to injury [208]. Adipocytes can behave in an immune-like way and sense inflammatory signals that consequently modify adipocyte functions and modulate immune responses [209]. The serum concentration of adipokines, such as leptin and resistin, are elevated in CKD [150] as a result of declined renal elimination, but also as a result of increased production by stimulated adipocytes in the uremic milieu [210].

High leptin levels and the associated inflammation contribute to the initiation and development of renal disease [211,212]. Leptin inhibits PMNL chemotaxis in a reversible manner and attenuates the activation of PMNL oxidative burst [213]. In humans, resistin is expressed mainly by macrophages in the visceral white adipose tissue [214] as well as in PMNLs and monocytes [215]. Resistin directly inhibits bacterial killing in neutrophils [216]. It inhibits PMNL chemotaxis and attenuates the stimulation of PMNL oxidative burst [217]. Resistin is stored in PMNL granules and released after stimulation with inflammatory mediators [218]. Because resistin stimulates the chemotaxis of CD4-positve lymphocytes [219], PMNLs may attract lymphocytes to the site of inflammation while they attenuate their own functions.

### 8.4. Posttranslational Modifications

The posttranslational modifications in the uremic milieu may result in changed functions of enzymes, cofactors, hormones, low-density lipoproteins, antibodies, receptors, and transport proteins.

#### 8.4.1. Carbamoylation

Protein carbamoylation is a non-enzymatic post-translational modification resulting from the binding of free amino groups of proteins to isocyanic acid originating from the dissociation of urea or from the catabolism of thiocyanate catalyzed by MPO. Of note, in the literature, the term “carbamylation” is often used instead of “carbamoylation”. However, carbamylation refers to a different chemical reaction, namely the reversible interaction of CO_2_ with α and ε-amino groups of proteins.

The mechanisms and consequences of carbamoylation have recently been reviewed by Delanghe et al. [220]. Carbamoylated proteins are associated with atherosclerosis, lipid metabolism, and renal fibrosis. They also contribute to several aspects of immune system dysfunction, e.g., inhibition of the classical complement pathway, reduced oxidative PMNL burst, and formation of anti-carbamoylated protein antibodies. In ESRD, protein carbamoylation is associated with mortality [221]. PMNLs from peritoneal dialysis patients express carbamoylated proteins in their cytoplasm and on their cell surface, suggesting that posttranslational modifications of proteins by urea-derived cyanate may contribute to PMNL dysfunction in patients with renal disease [222]. Carbamoylation of collagen leads to the activation of PMNLs and disturbs the remodeling of the extracellular matrix, contributing to the pathophysiology of renal failure [223]. Carbamoylation may result in homocitrulline bound to serum proteins, predicting the increased risk for CVD in ESRD patients, indicating a link between uremia, inflammation, and atherosclerosis [221].

In contrast to carbamoylation and carbamylation, carbonylation is the irreversible modification of proteins with carbonyl derivatives, i.e., aldehydes and ketones, and occurs in chronic uremia because of “carbonyl stress”. Carbonyl reactive species mainly modify albumin. Carbonylated albumin may cause a significant rise of adhesion molecule expression in endothelial cells and thereby contribute to uremic atherosclerosis [224].

#### 8.4.2. Advanced Glycation End Products (AGEs)

AGEs are formed by non-enzymatic glycation between reducing sugars and amino acids, lipids, or DNA. They arise not only in the presence of hyperglycemia, but also in diseases with high levels of inflammation, such as CKD, where increased AGE concentrations result from increased formation as well as from decreased renal clearance. AGE levels are twice as high in patients with ESRD, compared with those in patients with diabetes mellitus without renal disease [225]. Stinghen et al. have previously reviewed the role of AGEs as uremic toxins [226]. AGEs exert their harmful effects by binding to their cell surface receptor, RAGE [227].

Compared to unmodified proteins, proteins modified with glucose in vitro increase PMNL chemotaxis and the stimulation of glucose uptake by PMNLs [228]. PMNL apoptosis is enhanced in the presence of glucose-modified serum proteins. Albumin modified with specific AGE compounds has an activating, potentially pro- atherogenic effect on leukocyte responses [229]. AGEs are chemotactic for human monocytes. Monocyte migration across intact endothelial cell monolayers can be triggered by sub-endothelial AGEs [230]. AGEs activate TNFα and IL-1β secretion by peritoneal macrophages in peritoneal dialysis patients and thereby contribute to the disturbed permeability of the peritoneal membrane in long-term PD patients [231]. Glycation of collagen in uremia may contribute to the disturbed host defense in CKD patients by increasing PMNL adhesion to collagen surfaces via the AGE receptor [232].

#### 8.4.3. Oxidative Modifications

AOPPs are markers of phagocyte-derived oxidative stress. AOPPs also interact with RAGE, suggesting that different oxidation products exert their biological effects via common signal transduction pathways [233]. As uremic toxins with pro-inflammatory effects, they activate the oxidative burst in PMNLs and monocytes [234]. In pre-dialysis patients, AOPPs are generally formed by MPO-independent oxidation mechanisms, whereas in HD patients, AOPPs are primarily produced from MPO released by activated PMNLs [235]. ROS produced by activated PMNLs can modify serum proteins. In turn, these modified proteins activate PMNLs [236]. Therefore, the local PMNL-initiated oxidative alterations of serum proteins may be a general autocrine and paracrine pro-inflammatory mechanism enhancing PMNL activation and accumulation at the site of inflammation [236].

High levels of oxidized low-density lipoproteins contribute to the increased risk for atherosclerosis in patients with renal failure. Oxidized low-density lipoprotein is associated with activated monocytes and macrophages [237] and may directly activate T cells and induce apoptosis [238].

Albumin is the most important antioxidant in human serum. As a result of the higher susceptibility to proteases induced by oxidative stress in diabetic and ESRD patients, albumin can be fragmented [239]. On the other hand, oxidation of albumin contributes to the development of oxidative stress in HD patients [240].

### 8.5. High-Density Lipoprotein (HDL)

High-density lipoprotein (HDL) exerts a variety of essential biological functions [241]. Besides the classical role in reverse cholesterol transport [242], HDL has strong anti-oxidative, anti-inflammatory, and anti-thrombotic effects [243], contributing to both cardio-protection [244] and immuno-regulation [245,246]. PMNLs have binding sites for HDL [247] and their major apolipoprotein constituent ApoA-I [248]. HDL and Apo A-I significantly reduce CD11b surface expression on activated PMNLs [249] and monocytes [250] and decrease PMNL chemotactic movement [249]. Apo A-I inhibits PMNL adhesion, oxidative burst, and degranulation [251]. Inflammasome activation, which promotes NET formation in atherosclerotic plaques and enhances atherogenesis, is suppressed by cholesterol efflux to HDL [252]. Furthermore, reconstituted HDL inhibits the activation of human leukocytes in a whole blood assay as well as on monocyte-derived human dendritic cells [253].

In inflammatory diseases such as CKD, diabetes, and rheumatoid arthritis, HDL is qualitatively altered and loses its anti-inflammatory properties [254,255,256]. In CKD, these changes are primarily caused by uremic toxicity. The accumulation of the uremic toxin SDMA in HDL of CKD patients adds to its adverse effects [257]. The amount of serum amyloid A in the HDL particle from uremic patients inversely correlates with its anti-inflammatory effect [258]. Post-translational modifications such as glycation and carbamoylation give rise to modified HDL, which by itself contributes to uremic toxicity [259].

Studies on the effect of HDL from HD patients on vascular smooth muscle cells [260] and monocytes and dendritic cells [258] found decreased or eradicated anti-inflammatory features. Whereas HDL from healthy subjects reduces the production of inflammatory cytokines by peripheral monocytes, HDL isolated from CKD patients had no such effect [258]. We have shown that HDL from CKD and HD patients significantly attenuated PMNL apoptosis, whereas HDL from healthy subjects had no effect [261]. HDL isolated from healthy subjects diminished the activation of CD11b surface expression, whilst HDL from CKD and HD patients had no such impact, indicating that HDL may contribute to the systemic inflammation in uremic patients by modulating PMNL functions [261].

### 8.6. Hydrogen Sulfide (H_2_S)

Hydrogen sulfide (H_2_S) is a toxic gas. However, it is also endogenously produced in very small amounts. Therefore, it belongs to the family of gasotransmitters together with nitric oxide and carbon monoxide. H_2_S has several protective effects on the physiology of the kidney, increases GFR, and shows anti-inflammatory, anti-oxidative, and anti-apoptotic properties [262]. The role of H_2_S on important renal functions has been previously reviewed [263]. H_2_S affects the renin secretion from juxtaglomerular cells and consequently regulates blood pressure. When the RAS is over-activated, H_2_S downregulates cAMP, a modulator of renin release, by inhibiting adenylate cyclase. In diabetic nephropathy, H_2_S attenuates ROS production, activates AMP-activated protein kinase, and stimulates NO-formation.

In patients with a reduced kidney function, H_2_S levels are decreased [264]. This is frequently associated with atherosclerosis, hypertension, myocardial infarction, and diabetes. After a single HD session, H_2_S concentration increases, implying that uremic toxins that inhibit H_2_S -producing enzymes are removed [265]. The levels of lanthionine, a side-product of H_2_S biosynthesis, are significantly elevated in uremia. Vigorito et al. recently showed that lanthionine inhibits H_2_S release by reducing protein content and glutathionylation of the transsulfuration enzyme cystathionine-beta-synthase [266].

There are diverging findings regarding the effects of H_2_S on the immune system [267]. In several models of inflammation (sepsis, endotoxic, and hemorrhagic shock), H_2_S levels were increased, indicating that H_2_S has pro-inflammatory effects [268,269,270]. Rinaldi et al. [271] found decreased PMNL apoptosis under the effect of H_2_S, released by NaHS, *in vitro*. Furthermore, Spiller et al. [272] showed that NaHS stimulates PMNL migration to the side of infection in septic mice.

However, other studies suggested anti-inflammatory features of H_2_S. H_2_S has a prominent role in the resolution of inflammation (reviewed in [273]). H_2_S modulates inflammatory reactions at the level of leukocytes and endothelium [274]. H_2_S, released by the H_2_S donor GYY41137, decreased PMNL chemotaxis and attenuated the production of ROS in a mice model of lipopolysaccharide-induced acute lung injury [275]. The opposite and apparently contradictory results of in vitro studies might be explained by different kinetics of H_2_S release from the various H_2_S donors and the resulting different local H_2_S concentrations.

## 9. Conclusions

CVD and infections are directly or indirectly linked to a disturbed immune defense and contribute to the high incidence of morbidity and mortality of patients with reduced kidney function. In uremia, both defective renal metabolic activities and impaired glomerular filtration resulting in the accumulation of uremic toxins interfere with the immune system.

## References

[1] Zemaitis M.R., Foris L.A., Chandra S., Bashir K. (2020). Uremia.

[2] (2013). Chapter 1: Definition and classification of CKD. Kidney Int. Suppl..

[3] Hill N.R., Fatoba S.T., Oke J.L., Hirst J.A., O’Callaghan C.A., Lasserson D.S., Hobbs F.D. (2016). Global Prevalence of Chronic Kidney Disease—A Systematic Review and Meta-Analysis. PLoS ONE.

[4] Webster A.C., Nagler E.V., Morton R.L., Masson P. (2017). Chronic Kidney Disease. Lancet.

[5] Zou Z., Cini K., Dong B., Ma Y., Ma J., Burgner D.P., Patton G.C. (2020). Time Trends in Cardiovascular Disease Mortality Across the BRICS: An Age-Period-Cohort Analysis of Key Nations With Emerging Economies Using the Global Burden of Disease Study 2017. Circulation.

[6] Gansevoort R.T., Correa-Rotter R., Hemmelgarn B.R., Jafar T.H., Heerspink H.J., Mann J.F., Matsushita K., Wen C.P. (2013). Chronic kidney disease and cardiovascular risk: Epidemiology, mechanisms, and prevention. Lancet.

[7] Kato S., Chmielewski M., Honda H., Pecoits-Filho R., Matsuo S., Yuzawa Y., Tranaeus A., Stenvinkel P., Lindholm B. (2008). Aspects of immune dysfunction in end-stage renal disease. Clin. J. Am. Soc. Nephrol..

[8] Tonelli M., Wiebe N., Culleton B., House A., Rabbat C., Fok M., McAlister F., Garg A.X. (2006). Chronic kidney disease and mortality risk: A systematic review. J. Am. Soc. Nephrol..

[9] Kade G., Lubas A., Bodnar L., Szczylik C., Wankowicz Z. (2012). Malignant tumors in patients with end stage renal failure undergoing renal replacement therapy. Contemp. Oncol. (Pozn).

[10] Malyszko J., Kozlowski L., Kozlowska K., Malyszko M., Malyszko J. (2017). Cancer and the kidney: Dangereoux liasons or price paid for the progress in medicine?. Oncotarget.

[11] Maisonneuve P., Agodoa L., Gellert R., Stewart J.H., Buccianti G., Lowenfels A.B., Wolfe R.A., Jones E., Disney A.P., Briggs D. (1999). Cancer in patients on dialysis for end-stage renal disease: An international collaborative study. Lancet.

[12] Thompson S., James M., Wiebe N., Hemmelgarn B., Manns B., Klarenbach S., Tonelli M., Alberta Kidney Disease N. (2015). Cause of Death in Patients with Reduced Kidney Function. J. Am. Soc. Nephrol..

[13] White W.E., Yaqoob M.M., Harwood S.M. (2015). Aging and uremia: Is there cellular and molecular crossover?. World J. Nephrol..

[14] Kooman J.P., Broers N.J., Usvyat L., Thijssen S., van der Sande F.M., Cornelis T., Levin N.W., Leunissen K.M., Kotanko P. (2013). Out of control: Accelerated aging in uremia. Nephrol. Dial. Transplant..

[15] Di Lullo L., House A., Gorini A., Santoboni A., Russo D., Ronco C. (2015). Chronic kidney disease and cardiovascular complications. Heart Fail. Rev..

[16] Ryan T.P., Fisher S.G., Elder J.L., Winters P.C., Beckett W., Tacci J., Sloand J.A. (2009). Increased Cardiovascular Risk Associated with Reduced Kidney Function. Am. J. Nephrol..

[17] Cozzolino M., Mangano M., Stucchi A., Ciceri P., Conte F., Galassi A. (2018). Cardiovascular disease in dialysis patients. Nephrol. Dial. Transplant..

[18] Sarnak M.J., Jaber B.L. (2000). Mortality caused by sepsis in patients with end-stage renal disease compared with the general population. Kidney Int..

[19] Mansur A., Mulwande E., Steinau M., Bergmann I., Popov A.F., Ghadimi M., Beissbarth T., Bauer M., Hinz J. (2015). Chronic kidney disease is associated with a higher 90-day mortality than other chronic medical conditions in patients with sepsis. Sci. Rep..

[20] Powe N.R., Jaar B., Furth S.L., Hermann J., Briggs W. (1999). Septicemia in dialysis patients: Incidence, risk factors, and prognosis. Kidney Int..

[21] James M.T., Laupland K.B., Tonelli M., Manns B.J., Culleton B.F., Hemmelgarn B.R. (2008). Risk of bloodstream infection in patients with chronic kidney disease not treated with dialysis. Arch. Intern. Med..

[22] Eleftheriadis T., Antoniadi G., Liakopoulos V., Kartsios C., Stefanidis I. (2007). Disturbances of acquired immunity in hemodialysis patients. Semin. Dial..

[23] Kurts C., Panzer U., Anders H.J., Rees A.J. (2013). The immune system and kidney disease: Basic concepts and clinical implications. Nat. Rev. Immunol..

[24] Lahoz-Beneytez J., Elemans M., Zhang Y., Ahmed R., Salam A., Block M., Niederalt C., Asquith B., Macallan D. (2016). Human neutrophil kinetics: Modeling of stable isotope labeling data supports short blood neutrophil half-lives. Blood.

[25] Kolaczkowska E., Kubes P. (2013). Neutrophil recruitment and function in health and inflammation. Nat. Rev. Immunol..

[26] Haag-Weber M., Hörl W.H. (1996). Dysfunction of polymorphonuclear leukocytes in uremia. Semin. Nephrol..

[27] Chonchol M. (2006). Neutrophil dysfunction and infection risk in end-stage renal disease. Semin. Dial..

[28] Kim J.K., Hong C.W., Park M.J., Song Y.R., Kim H.J., Kim S.G. (2017). Increased Neutrophil Extracellular Trap Formation in Uremia Is Associated with Chronic Inflammation and Prevalent Coronary Artery Disease. J. Immunol. Res..

[29] Bratton D.L., Henson P.M. (2011). Neutrophil clearance: When the party is over, clean-up begins. Trends Immunol..

[30] Filep J.G., El Kebir D. (2009). Neutrophil apoptosis: A target for enhancing the resolution of inflammation. J. Cell Biochem..

[31] Haag-Weber M., Hörl W.H. (1992). Calcium-dependent neutrophil activation. Contrib. Nephrol..

[32] Hörl W.H., Haag-Weber M., Mai B., Massry S.G. (1995). Verapamil reverses abnormal [Ca2+]i and carbohydrate metabolism of PMNL of dialysis patients. Kidney Int..

[33] Lucas M., Diaz P. (2001). Thapsigargin-induced calcium entry and apoptotic death of neutrophils are blocked by activation of protein kinase C. Pharmacology.

[34] Hu T.H., Bei L., Huang Y.F., Shen X. (1999). The relationship between fMLP induced neutrophil respiratory burst and the apoptosis of neutrophil. Shi Yan Sheng Wu Xue Bao.

[35] Kettritz R., Falk R.J., Jennette J.C., Gaido M.L. (1997). Neutrophil superoxide release is required for spontaneous and FMLP-mediated but not for TNF alpha-mediated apoptosis. J. Am. Soc. Nephrol..

[36] Cohen G., Raupachova J., Wimmer T., Deicher R., Horl W.H. (2008). The uraemic retention solute para-hydroxy-hippuric acid attenuates apoptosis of polymorphonuclear leukocytes from healthy subjects but not from haemodialysis patients. Nephrol. Dial. Transplant..

[37] Massry S., Smogorzewski M. (2001). Dysfunction of polymorphonuclear leukocytes in uremia: Role of parathyroid hormone. Kidney Int. Suppl..

[38] Hann J., Bueb J.L., Tolle F., Brechard S. (2020). Calcium signaling and regulation of neutrophil functions: Still a long way to go. J. Leukoc. Biol..

[39] Zawrotniak M., Rapala-Kozik M. (2013). Neutrophil extracellular traps (NETs)—formation and implications. Acta Biochim. Pol..

[40] Papayannopoulos V., Zychlinsky A. (2009). NETs: A new strategy for using old weapons. Trends Immunol..

[41] De Buhr N., von Kockritz-Blickwede M. (2016). How Neutrophil Extracellular Traps Become Visible. J. Immunol. Res..

[42] Kim J.K., Lee H.W., Joo N., Lee H.S., Song Y.R., Kim H.J., Kim S.G. (2019). Prognostic role of circulating neutrophil extracellular traps levels for long-term mortality in new end-stage renal disease patients. Clin. Immunol..

[43] Korabecna M., Tesar V. (2017). NETosis provides the link between activation of neutrophils on hemodialysis membrane and comorbidities in dialyzed patients. Inflamm. Res..

[44] Darrah E., Andrade F. (2012). NETs: The missing link between cell death and systemic autoimmune diseases?. Front. Immunol..

[45] Swain S.D., Rohn T.T., Quinn M.T. (2002). Neutrophil priming in host defense: Role of oxidants as priming agents. Antioxid. Redox Signal..

[46] Vogt K.L., Summers C., Chilvers E.R., Condliffe A.M. (2018). Priming and de-priming of neutrophil responses in vitro and in vivo. Eur. J. Clin. Invest..

[47] Klein J.B., McLeish K.R., Ward R.A. (1999). Transplantation, not dialysis, corrects azotemia-dependent priming of the neutrophil oxidative burst. Am. J. Kidney Dis..

[48] Chilvers E.R., Cadwallader K.A., Reed B.J., White J.F., Condliffe A.M. (2000). The function and fate of neutrophils at the inflamed site: Prospects for therapeutic intervention. J. R. Coll. Physicians Lond..

[49] El-Benna J., Hurtado-Nedelec M., Marzaioli V., Marie J.C., Gougerot-Pocidalo M.A., Dang P.M. (2016). Priming of the neutrophil respiratory burst: Role in host defense and inflammation. Immunol. Rev..

[50] Sela S., Shurtz-Swirski R., Cohen-Mazor M., Mazor R., Chezar J., Shapiro G., Hassan K., Shkolnik G., Geron R., Kristal B. (2005). Primed peripheral polymorphonuclear leukocyte: A culprit underlying chronic low-grade inflammation and systemic oxidative stress in chronic kidney disease. J. Am. Soc. Nephrol..

[51] Girndt M., Trojanowicz B., Ulrich C. (2020). Monocytes in Uremia. Toxins (Basel).

[52] Jeng Y., Lim P.S., Wu M.Y., Tseng T.Y., Chen C.H., Chen H.P., Wu T.K. (2017). Proportions of Proinflammatory Monocytes Are Important Predictors of Mortality Risk in Hemodialysis Patients. Mediators Inflamm..

[53] Verkade M.A., van Druningen C.J., Vaessen L.M., Hesselink D.A., Weimar W., Betjes M.G. (2007). Functional impairment of monocyte-derived dendritic cells in patients with severe chronic kidney disease. Nephrol. Dial. Transplant..

[54] Kim J.U., Kim M., Kim S., Nguyen T.T., Kim E., Lee S., Kim H. (2017). Dendritic Cell Dysfunction in Patients with End-stage Renal Disease. Immune Netw..

[55] Kitching A.R. (2014). Dendritic cells in progressive renal disease: Some answers, many questions. Nephrol. Dial. Transplant..

[56] Panzer U., Kurts C. (2010). T cell cross-talk with kidney dendritic cells in glomerulonephritis. J. Mol. Med..

[57] Betjes M.G. (2013). Immune cell dysfunction and inflammation in end-stage renal disease. Nat. Rev. Nephrol..

[58] Betjes M.G. (2020). Uremia-Associated Ageing of the Thymus and Adaptive Immune Responses. Toxins (Basel).

[59] Pahl M.V., Gollapudi S., Sepassi L., Gollapudi P., Elahimehr R., Vaziri N.D. (2010). Effect of end-stage renal disease on B-lymphocyte subpopulations, IL-7, BAFF and BAFF receptor expression. Nephrol. Dial. Transplant..

[60] Duni A., Liakopoulos V., Roumeliotis S., Peschos D., Dounousi E. (2019). Oxidative Stress in the Pathogenesis and Evolution of Chronic Kidney Disease: Untangling Ariadne’s Thread. Int. J. Mol. Sci..

[61] Dounousi E., Papavasiliou E., Makedou A., Ioannou K., Katopodis K.P., Tselepis A., Siamopoulos K.C., Tsakiris D. (2006). Oxidative stress is progressively enhanced with advancing stages of CKD. Am. J. Kidney Dis..

[62] Suvakov S., Jerotic D., Damjanovic T., Milic N., Pekmezovic T., Djukic T., Jelic-Ivanovic Z., Savic Radojevic A., Pljesa-Ercegovac M., Matic M. (2019). Markers of Oxidative Stress and Endothelial Dysfunction Predict Haemodialysis Patients Survival. Am. J. Nephrol..

[63] Morena M., Cristol J.P., Senecal L., Leray-Moragues H., Krieter D., Canaud B. (2002). Oxidative stress in hemodialysis patients: Is NADPH oxidase complex the culprit?. Kidney Int. Suppl..

[64] Libetta C., Sepe V., Esposito P., Galli F., Dal Canton A. (2011). Oxidative stress and inflammation: Implications in uremia and hemodialysis. Clin. Biochem..

[65] Ward R.A., McLeish K.R. (2004). Methylglyoxal: A stimulus to neutrophil oxygen radical production in chronic renal failure?. Nephrol. Dial. Transplant..

[66] Martini S., Nair V., Keller B.J., Eichinger F., Hawkins J.J., Randolph A., Boger C.A., Gadegbeku C.A., Fox C.S., Cohen C.D. (2014). Integrative biology identifies shared transcriptional networks in CKD. J. Am. Soc. Nephrol..

[67] Jerotic D., Matic M., Suvakov S., Vucicevic K., Damjanovic T., Savic-Radojevic A., Pljesa-Ercegovac M., Coric V., Stefanovic A., Ivanisevic J. (2019). Association of Nrf2, SOD2 and GPX1 Polymorphisms with Biomarkers of Oxidative Distress and Survival in End-Stage Renal Disease Patients. Toxins (Basel).

[68] Jofre R., Rodriguez-Benitez P., Lopez-Gomez J.M., Perez-Garcia R. (2006). Inflammatory syndrome in patients on hemodialysis. J. Am. Soc. Nephrol..

[69] Wann J.G., Hsu Y.H., Yang C.C., Lin C.S., Tai D.W., Chen J.S., Hsiao C.W., Chen C.F. (2007). Neutrophils in acidotic haemodialysed patients have lower intracellular pH and inflamed state. Nephrol. Dial. Transplant..

[70] Jha J.C., Ho F., Dan C., Jandeleit-Dahm K. (2018). A causal link between oxidative stress and inflammation in cardiovascular and renal complications of diabetes. Clin. Sci. (Lond.).

[71] Jha V., Garcia-Garcia G., Iseki K., Li Z., Naicker S., Plattner B., Saran R., Wang A.Y., Yang C.W. (2013). Chronic kidney disease: Global dimension and perspectives. Lancet.

[72] Perkovic V., Agarwal R., Fioretto P., Hemmelgarn B.R., Levin A., Thomas M.C., Wanner C., Kasiske B.L., Wheeler D.C., Groop P.H. (2016). Management of patients with diabetes and CKD: Conclusions from a “Kidney Disease: Improving Global Outcomes” (KDIGO) Controversies Conference. Kidney Int..

[73] Jankowska M., Cobo G., Lindholm B., Stenvinkel P. (2017). Inflammation and Protein-Energy Wasting in the Uremic Milieu. Contrib Nephrol.

[74] Yao Q., Axelsson J., Stenvinkel P., Lindholm B. (2004). Chronic systemic inflammation in dialysis patients: An update on causes and consequences. Asaio J..

[75] Helal I., Smaoui W., Hamida F.B., Ouniss M., Aderrahim E., Hedri H., Elyounsi F., Maiz H.B., Abdallah T.B., Kheder A. (2010). Cardiovascular risk factors in hemodialysis and peritoneal dialysis patients. Saudi J. Kidney Dis. Transpl..

[76] Leemans J.C., Kors L., Anders H.J., Florquin S. (2014). Pattern recognition receptors and the inflammasome in kidney disease. Nat. Rev. Nephrol..

[77] Takeuchi O., Akira S. (2010). Pattern recognition receptors and inflammation. Cell.

[78] Gollapudi P., Yoon J.W., Gollapudi S., Pahl M.V., Vaziri N.D. (2010). Leukocyte Toll-Like Receptor Expression in End-Stage Kidney Disease. Am. J. Nephrol..

[79] Baj Z., Zbrog Z., Szuflet A., Manka S., Bartnicki P., Majewska E. (2018). Basic inflammatory indices and chosen neutrophil receptors expression in chronic haemodialysed patients. Cent. Eur. J. Immunol..

[80] Shen H., Kreisel D., Goldstein D.R. (2013). Processes of sterile inflammation. J. Immunol..

[81] Komada T., Muruve D.A. (2019). The role of inflammasomes in kidney disease. Nat. Rev. Nephrol..

[82] Broz P., Dixit V.M. (2016). Inflammasomes: Mechanism of assembly, regulation and signalling. Nat. Rev. Immuno.l.

[83] Hung S.C., Kuo K.L., Peng C.H., Wu C.H., Lien Y.C., Wang Y.C., Tarng D.C. (2014). Volume overload correlates with cardiovascular risk factors in patients with chronic kidney disease. Kidney Int..

[84] Zoccali C., Moissl U., Chazot C., Mallamaci F., Tripepi G., Arkossy O., Wabel P., Stuard S. (2017). Chronic Fluid Overload and Mortality in ESRD. J. Am. Soc. Nephrol..

[85] Dekker M.J.E., van der Sande F.M., van den Berghe F., Leunissen K.M.L., Kooman J.P. (2018). Fluid Overload and Inflammation Axis. Blood Purif..

[86] Ulrich C., Wilke A., Schleicher N., Girndt M., Fiedler R. (2020). Hypervolemia-Induced Immune Disturbances Do Not Involve IL-1ss but IL-6 and IL-10 Activation in Haemodialysis Patients. Toxins (Basel).

[87] Mizushima N., Komatsu M. (2011). Autophagy: Renovation of cells and tissues. Cell.

[88] Kim J.K., Park M.J., Lee H.W., Lee H.S., Choi S.R., Song Y.R., Kim H.J., Park H.C., Kim S.G. (2018). The relationship between autophagy, increased neutrophil extracellular traps formation and endothelial dysfunction in chronic kidney disease. Clin. Immunol..

[89] Deretic V., Kimura T., Timmins G., Moseley P., Chauhan S., Mandell M. (2015). Immunologic manifestations of autophagy. J. Clin. Invest..

[90] Zhou R., Yazdi A.S., Menu P., Tschopp J. (2011). A role for mitochondria in NLRP3 inflammasome activation. Nature.

[91] Shi C.S., Shenderov K., Huang N.N., Kabat J., Abu-Asab M., Fitzgerald K.A., Sher A., Kehrl J.H. (2012). Activation of autophagy by inflammatory signals limits IL-1beta production by targeting ubiquitinated inflammasomes for destruction. Nat. Immunol..

[92] Martinez J., Cunha L.D., Park S., Yang M., Lu Q., Orchard R., Li Q.Z., Yan M., Janke L., Guy C. (2016). Noncanonical autophagy inhibits the autoinflammatory, lupus-like response to dying cells. Nature.

[93] Lee H.K., Lund J.M., Ramanathan B., Mizushima N., Iwasaki A. (2007). Autophagy-dependent viral recognition by plasmacytoid dendritic cells. Science.

[94] Lin T.A., Wu V.C., Wang C.Y. (2019). Autophagy in Chronic Kidney Diseases. Cells.

[95] Fougeray S., Pallet N. (2015). Mechanisms and biological functions of autophagy in diseased and ageing kidneys. Nat. Rev. Nephrol..

[96] Jin Y., Tanaka A., Choi A.M., Ryter S.W. (2012). Autophagic proteins: New facets of the oxygen paradox. Autophagy.

[97] Chen W.T., Hung K.C., Wen M.S., Hsu P.Y., Chen T.H., Wang H.D., Fang J.T., Shie S.S., Wang C.Y. (2013). Impaired leukocytes autophagy in chronic kidney disease patients. Cardiorenal. Med..

[98] Ren C., Zhang H., Wu T.T., Yao Y.M. (2017). Autophagy: A Potential Therapeutic Target for Reversing Sepsis-Induced Immunosuppression. Front. Immunol..

[99] Tecklenborg J., Clayton D., Siebert S., Coley S.M. (2018). The role of the immune system in kidney disease. Clin. Exp. Immunol..

[100] Willows J., Brown M., Sheerin N.S. (2020). The role of complement in kidney disease. Clin. Med. (Lond.).

[101] Riedl M., Noone D.G., Khan M.A., Pluthero F.G., Kahr W.H.A., Palaniyar N., Licht C. (2017). Complement Activation Induces Neutrophil Adhesion and Neutrophil-Platelet Aggregate Formation on Vascular Endothelial Cells. Kidney Int. Rep..

[102] Thurman J.M. (2015). Complement in kidney disease: Core curriculum 2015. Am. J. Kidney Dis..

[103] McCullough J.W., Renner B., Thurman J.M. (2013). The role of the complement system in acute kidney injury. Semin. Nephrol..

[104] Franzin R., Stasi A., Fiorentino M., Stallone G., Cantaluppi V., Gesualdo L., Castellano G. (2020). Inflammaging and Complement System: A Link Between Acute Kidney Injury and Chronic Graft Damage. Front. Immunol..

[105] De Borst M.H. (2019). Interaction between inflammation, mineral metabolism and the renin-angiotensin system: Implications for cardiorenal outcomes in chronic kidney disease. Nephrol. Dial. Transplant..

[106] Crowley S.D., Rudemiller N.P. (2017). Immunologic Effects of the Renin-Angiotensin System. J. Am. Soc. Nephrol..

[107] Trojanowicz B., Ulrich C., Kohler F., Bode V., Seibert E., Fiedler R., Girndt M. (2017). Monocytic angiotensin-converting enzyme 2 relates to atherosclerosis in patients with chronic kidney disease. Nephrol. Dial. Transplant..

[108] Ulrich C., Heine G.H., Garcia P., Reichart B., Georg T., Krause M., Kohler H., Girndt M. (2006). Increased expression of monocytic angiotensin-converting enzyme in dialysis patients with cardiovascular disease. Nephrol. Dial. Transplant..

[109] Trojanowicz B., Imdahl T., Ulrich C., Fiedler R., Girndt M. (2019). Circulating miR-421 Targeting Leucocytic Angiotensin Converting Enzyme 2 Is Elevated in Patients with Chronic Kidney Disease. Nephron.

[110] Jurewicz M., McDermott D.H., Sechler J.M., Tinckam K., Takakura A., Carpenter C.B., Milford E., Abdi R. (2007). Human T and natural killer cells possess a functional renin-angiotensin system: Further mechanisms of angiotensin II-induced inflammation. J. Am. Soc. Nephrol..

[111] El Bekay R., Alvarez M., Monteseirin J., Alba G., Chacon P., Vega A., Martin-Nieto J., Jimenez J., Pintado E., Bedoya F.J. (2003). Oxidative stress is a critical mediator of the angiotensin II signal in human neutrophils: Involvement of mitogen-activated protein kinase, calcineurin, and the transcription factor NF-kappaB. Blood.

[112] Martinez F., Pallet N. (2014). When erythropoietin meddles in immune affairs. J. Am. Soc. Nephrol..

[113] Gavish R., Watad S., Ben-Califa N., Goldberg O.J., Haskin O., Davidovits M., Koren G., Falush Y., Neumann D., Krause I. (2018). Response to erythropoietin in pediatric patients with chronic kidney disease: Insights from an in vitro bioassay. Pediatr. Nephrol..

[114] Eschbach J.W., Egrie J.C., Downing M.R., Browne J.K., Adamson J.W. (1987). Correction of the anemia of end-stage renal disease with recombinant human erythropoietin. Results of a combined phase I and II clinical trial. N. Engl. J. Med..

[115] Macdougall I.C., Cooper A.C. (2002). Erythropoietin resistance: The role of inflammation and pro-inflammatory cytokines. Nephrol. Dial. Transplant..

[116] Patruta S.I., Hörl W.H. (1999). Iron and infection. Kidney Int. Suppl..

[117] Yen C.L., Lin Y.S., Lu Y.A., Lee H.F., Lee C.C., Tung Y.C., Kuo G., Wu L.S., Tian Y.C., Chu P.H. (2019). Intravenous iron supplementation does not increase infectious disease risk in hemodialysis patients: A nationwide cohort-based case-crossover study. BMC Nephrol..

[118] Lisowska K.A., Jasiulewicz A., Bryl E., Witkowski J.M. (2011). Erythropoietin as an Immunomodulating Agent. Nephro-Urol Mon..

[119] Sela S., Shurtz-Swirski R., Sharon R., Manaster J., Chezar J., Shkolnik G., Shapiro G., Shasha S.M., Merchav S., Kristal B. (2001). The polymorphonuclear leukocyte--a new target for erythropoietin. Nephron.

[120] Lisowska K.A., Debska-Slizien A., Bryl E., Rutkowski B., Witkowski J.M. (2010). Erythropoietin receptor is expressed on human peripheral blood T and B lymphocytes and monocytes and is modulated by recombinant human erythropoietin treatment. Artif. Organs.

[121] Rocchetta F., Solini S., Mister M., Mele C., Cassis P., Noris M., Remuzzi G., Aiello S. (2011). Erythropoietin enhances immunostimulatory properties of immature dendritic cells. Clin. Exp. Immunol..

[122] Cravedi P., Manrique J., Hanlon K.E., Reid-Adam J., Brody J., Prathuangsuk P., Mehrotra A., Heeger P.S. (2014). Immunosuppressive effects of erythropoietin on human alloreactive T cells. J. Am. Soc. Nephrol..

[123] Begum S., Latunde-Dada G.O. (2019). Anemia of Inflammation with An Emphasis on Chronic Kidney Disease. Nutrients.

[124] Atanasiu V., Manolescu B., Stoian I. (2006). Hepcidin the link between inflammation and anemia in chronic renal failure. Rom. J. Intern. Med..

[125] Deicher R., Horl W.H. (2006). New insights into the regulation of iron homeostasis. Eur. J. Clin. Invest..

[126] Saneela S., Iqbal R., Raza A., Qamar M.F. (2019). Hepcidin: A key regulator of iron. J. Pak. Med. Assoc..

[127] Ashby D.R., Gale D.P., Busbridge M., Murphy K.G., Duncan N.D., Cairns T.D., Taube D.H., Bloom S.R., Tam F.W., Chapman R.S. (2009). Plasma hepcidin levels are elevated but responsive to erythropoietin therapy in renal disease. Kidney Int..

[128] Lee S.W., Kim J.M., Lim H.J., Hwang Y.H., Kim S.W., Chung W., Oh K.H., Ahn C., Lee K.B., Sung S.A. (2017). Serum hepcidin may be a novel uremic toxin, which might be related to erythropoietin resistance. Sci. Rep..

[129] Reichel H., Recker A., Deppisch R., Stier E., Ritz E. (1992). 25-Hydroxyvitamin D3 metabolism in vitro by mononuclear cells from hemodialysis patients. Nephron.

[130] Schomig M., Ritz E. (2000). Management of disturbed calcium metabolism in uraemic patients: 1. Use of vitamin D metabolites. Nephrol. Dial. Transplant..

[131] Glorieux G., Vanholder R. (2001). Blunted response to vitamin D in uremia. Kidney Int Suppl.

[132] Lang C.L., Wang M.H., Chiang C.K., Lu K.C. (2014). Vitamin D and the Immune System from the Nephrologist’s Viewpoint. ISRN Endocrinol..

[133] Shroff R., Wan M., Rees L. (2011). Can vitamin D slow down the progression of chronic kidney disease?. Pediatr. Nephrol..

[134] Szeto F.L., Reardon C.A., Yoon D., Wang Y., Wong K.E., Chen Y., Kong J., Liu S.Q., Thadhani R., Getz G.S. (2012). Vitamin d receptor signaling inhibits atherosclerosis in mice. Mol. Endocrinol..

[135] Carvalho J.T.G., Schneider M., Cuppari L., Grabulosa C.C., Danilo T.A., Redublo B.M.Q., Marcelo C.B., Cendoroglo M., Maria Moyses R., Dalboni M.A. (2017). Cholecalciferol decreases inflammation and improves vitamin D regulatory enzymes in lymphocytes in the uremic environment: A randomized controlled pilot trial. PLoS ONE.

[136] Kaur G., Singh J., Kumar J. (2019). Vitamin D and cardiovascular disease in chronic kidney disease. Pediatr. Nephrol..

[137] Wahl P., Wolf M. (2012). FGF23 in chronic kidney disease. Adv. Exp. Med. Biol..

[138] Vogt I., Haffner D., Leifheit-Nestler M. (2019). FGF23 and Phosphate-Cardiovascular Toxins in CKD. Toxins (Basel).

[139] Czaya B., Faul C. (2019). The Role of Fibroblast Growth Factor 23 in Inflammation and Anemia. Int. J. Mol. Sci..

[140] Isakova T., Cai X., Lee J., Xie D., Wang X., Mehta R., Allen N.B., Scialla J.J., Pencina M.J., Anderson A.H. (2018). Longitudinal FGF23 Trajectories and Mortality in Patients with CKD. J. Am. Soc. Nephrol..

[141] Pichler G., Haller M.C., Kainz A., Wolf M., Redon J., Oberbauer R. (2017). Prognostic value of bone- and vascular-derived molecular biomarkers in hemodialysis and renal transplant patients: A systematic review and meta-analysis. Nephrol. Dial. Transplant..

[142] Takashi Y., Wakino S., Minakuchi H., Ishizu M., Kuroda A., Shima H., Tashiro M., Miya K., Okada K., Minakuchi J. (2020). Circulating FGF23 is not associated with cardiac dysfunction, atherosclerosis, infection or inflammation in hemodialysis patients. J Bone Miner. Metab..

[143] Memmos E., Sarafidis P., Pateinakis P., Tsiantoulas A., Faitatzidou D., Giamalis P., Vasilikos V., Papagianni A. (2019). Soluble Klotho is associated with mortality and cardiovascular events in hemodialysis. BMC Nephrol..

[144] Block G.A., Kilpatrick R.D., Lowe K.A., Wang W., Danese M.D. (2013). CKD-mineral and bone disorder and risk of death and cardiovascular hospitalization in patients on hemodialysis. Clin. J. Am. Soc. Nephrol..

[145] Duque E.J., Elias R.M., Moyses R.M.A. (2020). Parathyroid Hormone: A Uremic Toxin. Toxins (Basel).

[146] Deicher R., Kirsch B., Mullner M., Kaczirek K., Niederle B., Horl W.H. (2005). Impact of parathyroidectomy on neutrophil cytosolic calcium in chronic kidney disease patients: A prospective parallel group trial. J. Intern. Med..

[147] Smogorzewski M., Massry S.G. (2001). Defects in B-cell function and metabolism in uremia: Role of parathyroid hormone. Kidney Int. Suppl..

[148] Griveas I., Visvardis G., Papadopoulou D., Mitsopoulos E., Kyriklidou P., Manou E., Meimaridou D., Ginikopoulou E., Sakellariou G., Fleva A. (2005). Cellular immunity and levels of parathyroid hormone in uremic patients receiving hemodialysis. Ren. Fail..

[149] Vanholder R., Argiles A., Baurmeister U., Brunet P., Clark W., Cohen G., De Deyn P.P., Deppisch R., Descamps-Latscha B., Henle T. (2001). Uremic toxicity: Present state of the art. Int. J. Artif. Organs.

[150] Vanholder R., De Smet R., Glorieux G., Argiles A., Baurmeister U., Brunet P., Clark W., Cohen G., De Deyn P.P., Deppisch R. (2003). Review on uremic toxins: Classification, concentration, and interindividual variability. Kidney Int..

[151] Duranton F., Cohen G., De Smet R., Rodriguez M., Jankowski J., Vanholder R., Argiles A. (2012). Normal and pathologic concentrations of uremic toxins. J. Am. Soc. Nephrol..

[152] Schepers E., Glorieux G., Vanholder R. (2010). The gut: The forgotten organ in uremia?. Blood Purif..

[153] Meijers B., Glorieux G., Poesen R., Bakker S.J. (2014). Nonextracorporeal methods for decreasing uremic solute concentration: A future way to go?. Semin. Nephrol..

[154] Glorieux G., Gryp T., Perna A. (2020). Gut-Derived Metabolites and Their Role in Immune Dysfunction in Chronic Kidney Disease. Toxins (Basel).

[155] Perna A.F., Glorieux G., Zacchia M., Trepiccione F., Capolongo G., Vigorito C., Anishchenko E., Ingrosso D. (2019). The role of the intestinal microbiota in uremic solute accumulation: A focus on sulfur compounds. J. Nephrol..

[156] Maynard C.L., Elson C.O., Hatton R.D., Weaver C.T. (2012). Reciprocal interactions of the intestinal microbiota and immune system. Nature.

[157] Wang X., Yang S., Li S., Zhao L., Hao Y., Qin J., Zhang L., Zhang C., Bian W., Zuo L. (2020). Aberrant gut microbiota alters host metabolome and impacts renal failure in humans and rodents. Gut.

[158] Ramezani A., Massy Z.A., Meijers B., Evenepoel P., Vanholder R., Raj D.S. (2016). Role of the Gut Microbiome in Uremia: A Potential Therapeutic Target. Am. J. Kidney Dis..

[159] Popkov V.A., Silachev D.N., Zalevsky A.O., Zorov D.B., Plotnikov E.Y. (2019). Mitochondria as a Source and a Target for Uremic Toxins. Int. J. Mol. Sci..

[160] Neirynck N., Vanholder R., Schepers E., Eloot S., Pletinck A., Glorieux G. (2013). An update on uremic toxins. Int. Urol. Nephrol..

[161] Vanholder R., Pletinck A., Schepers E., Glorieux G. (2018). Biochemical and Clinical Impact of Organic Uremic Retention Solutes: A Comprehensive Update. Toxins (Basel).

[162] Rroji M., Eloot S., Dhondt A., Van Biesen W., Glorieux G., Neirynck N., Vandennoortgate N., Liabeuf S., Massy Z., Vanholder R. (2016). Association of advanced age with concentrations of uraemic toxins in CKD. J. Nephrol..

[163] Espi M., Koppe L., Fouque D., Thaunat O. (2020). Chronic Kidney Disease-Associated Immune Dysfunctions: Impact of Protein-Bound Uremic Retention Solutes on Immune Cells. Toxins (Basel).

[164] Kim H.Y., Yoo T.H., Hwang Y., Lee G.H., Kim B., Jang J., Yu H.T., Kim M.C., Cho J.Y., Lee C.J. (2017). Indoxyl sulfate (IS)-mediated immune dysfunction provokes endothelial damage in patients with end-stage renal disease (ESRD). Sci. Rep..

[165] Pletinck A., Glorieux G., Schepers E., Cohen G., Gondouin B., Van Landschoot M., Eloot S., Rops A., Van de Voorde J., De Vriese A. (2013). Protein-bound uremic toxins stimulate crosstalk between leukocytes and vessel wall. J. Am. Soc. Nephrol..

[166] Schepers E., Meert N., Glorieux G., Goeman J., Van der Eycken J., Vanholder R. (2007). P-cresylsulphate, the main in vivo metabolite of p-cresol, activates leucocyte free radical production. Nephrol. Dial. Transplant..

[167] Azevedo M.L., Bonan N.B., Dias G., Brehm F., Steiner T.M., Souza W.M., Stinghen A.E., Barreto F.C., Elifio-Esposito S., Pecoits-Filho R. (2016). p-Cresyl sulfate affects the oxidative burst, phagocytosis process, and antigen presentation of monocyte-derived macrophages. Toxicol. Lett..

[168] Pawlak K., Domaniewski T., Mysliwiec M., Pawlak D. (2009). The kynurenines are associated with oxidative stress, inflammation and the prevalence of cardiovascular disease in patients with end-stage renal disease. Atherosclerosis.

[169] Barth M.C., Ahluwalia N., Anderson T.J., Hardy G.J., Sinha S., Alvarez-Cardona J.A., Pruitt I.E., Rhee E.P., Colvin R.A., Gerszten R.E. (2009). Kynurenic acid triggers firm arrest of leukocytes to vascular endothelium under flow conditions. J. Biol. Chem..

[170] Schepers E., Glorieux G., Dou L., Cerini C., Gayrard N., Louvet L., Maugard C., Preus P., Rodriguez-Ortiz M., Argiles A. (2010). Guanidino compounds as cause of cardiovascular damage in chronic kidney disease: An in vitro evaluation. Blood Purif..

[171] Tain Y.L., Hsu C.N. (2017). Toxic Dimethylarginines: Asymmetric Dimethylarginine (ADMA) and Symmetric Dimethylarginine (SDMA). Toxins (Basel).

[172] Shafi T., Hostetter T.H., Meyer T.W., Hwang S., Hai X., Melamed M.L., Banerjee T., Coresh J., Powe N.R. (2017). Serum Asymmetric and Symmetric Dimethylarginine and Morbidity and Mortality in Hemodialysis Patients. Am. J. Kidney Dis..

[173] Glorieux G., Hsu C.H., de Smet R., Dhondt A., van Kaer J., Vogeleere P., Lameire N., Vanholder R. (1998). Inhibition of calcitriol-induced monocyte CD14 expression by uremic toxins: Role of purines. J. Am. Soc. Nephrol..

[174] Cohen G., Raupachova J., Horl W.H. (2013). The uraemic toxin phenylacetic acid contributes to inflammation by priming polymorphonuclear leucocytes. Nephrol. Dial. Transplant..

[175] Schepers E., Glorieux G., Jankowski V., Dhondt A., Jankowski J., Vanholder R. (2010). Dinucleoside polyphosphates: Newly detected uraemic compounds with an impact on leucocyte oxidative burst. Nephrol. Dial. Transplant..

[176] Okado A., Kawasaki Y., Hasuike Y., Takahashi M., Teshima T., Fujii J., Taniguchi N. (1996). Induction of apoptotic cell death by methylglyoxal and 3-deoxyglucosone in macrophage-derived cell lines. Biochem. Biophys. Res. Commun..

[177] Nakayama M., Nakayama K., Zhu W.J., Shirota Y., Terawaki H., Sato T., Kohno M., Ito S. (2008). Polymorphonuclear leukocyte injury by methylglyoxal and hydrogen peroxide: A possible pathological role for enhanced oxidative stress in chronic kidney disease. Nephrol. Dial. Transplant..

[178] Hannam-Harris A.C., Gordon J., Smith J.L. (1980). Immunoglobulin synthesis by neoplastic B lymphocytes: Free light chain synthesis as a marker of B cell differentiation. J. Immunol..

[179] Hutchison C.A., Harding S., Hewins P., Mead G.P., Townsend J., Bradwell A.R., Cockwell P. (2008). Quantitative assessment of serum and urinary polyclonal free light chains in patients with chronic kidney disease. Clin. J. Am. Soc. Nephrol..

[180] Cohen G., Rudnicki M., Schmaldienst S., Horl W.H. (2002). Effect of dialysis on serum/plasma levels of free immunoglobulin light chains in end-stage renal disease patients. Nephrol. Dial. Transplant..

[181] Hutchison C.A., Cockwell P., Reid S., Chandler K., Mead G.P., Harrison J., Hattersley J., Evans N.D., Chappell M.J., Cook M. (2007). Efficient removal of immunoglobulin free light chains by hemodialysis for multiple myeloma: In vitro and in vivo studies. J. Am. Soc. Nephrol..

[182] Kirsch A.H., Lyko R., Nilsson L.G., Beck W., Amdahl M., Lechner P., Schneider A., Wanner C., Rosenkranz A.R., Krieter D.H. (2017). Performance of hemodialysis with novel medium cut-off dialyzers. Nephrol. Dial. Transplant..

[183] Cohen G., Haag-Weber M., Mai B., Deicher R., Horl W.H. (1995). Effect of immunoglobulin light chains from hemodialysis and continuous ambulatory peritoneal dialysis patients on polymorphonuclear leukocyte functions. J. Am. Soc. Nephrol..

[184] Cohen G., Rudnicki M., Deicher R., Horl W.H. (2003). Immunoglobulin light chains modulate polymorphonuclear leucocyte apoptosis. Eur. J. Clin. Invest..

[185] Frey S.K., Nagl B., Henze A., Raila J., Schlosser B., Berg T., Tepel M., Zidek W., Weickert M.O., Pfeiffer A.F. (2008). Isoforms of retinol binding protein 4 (RBP4) are increased in chronic diseases of the kidney but not of the liver. Lipids Health Dis..

[186] Chen C.H., Hsieh T.J., Lin K.D., Lin H.Y., Lee M.Y., Hung W.W., Hsiao P.J., Shin S.J. (2012). Increased unbound retinol-binding protein 4 concentration induces apoptosis through receptor-mediated signaling. J. Biol. Chem..

[187] Farjo K.M., Farjo R.A., Halsey S., Moiseyev G., Ma J.X. (2012). Retinol-binding protein 4 induces inflammation in human endothelial cells by an NADPH oxidase- and nuclear factor kappa B-dependent and retinol-independent mechanism. Mol. Cell. Biol..

[188] Cohen G., Horl W.H. (2004). Retinol binding protein isolated from acute renal failure patients inhibits polymorphonuclear leucocyte functions. Eur. J. Clin. Invest..

[189] Wang J., Barke R.A., Ma J., Charboneau R., Roy S. (2008). Opiate abuse, innate immunity, and bacterial infectious diseases. Arch. Immunol. Ther. Exp. (Warsz).

[190] Zoccali C., Ciccarelli M., Mallamaci F., Maggiore Q., Lotti M., Zucchelli G.C. (1987). Plasma met-enkephalin and leu-enkephalin in chronic renal failure. Nephrol. Dial. Transplant..

[191] Pasnik J., Tchorzewski H., Baj Z., Luciak M., Tchorzewski M. (1999). Priming effect of met-enkephalin and beta-endorphin on chemiluminescence, chemotaxis and CD11b molecule expression on human neutrophils in vitro. Immunol. Lett..

[192] Wieder L. (2018). Effects of Met-Enkephalin, Neuropeptide-Y and Endothelin-1 on Essential Functions of Polymorphonuclear Leukocytes (PMNLs). Ph.D. Thesis.

[193] Deshmukh S., Phillips B.G., O’Dorisio T., Flanigan M.J., Lim V.S. (2005). Hormonal responses to fasting and refeeding in chronic renal failure patients. Am. J. Physiol. Endocrinol. Metab..

[194] Zoccali C., Mallamaci F., Tripepi G., Benedetto F.A., Parlongo S., Cutrupi S., Iellamo D., Bonanno G., Rapisarda F., Fatuzzo P. (2003). Prospective study of neuropeptide y as an adverse cardiovascular risk factor in end-stage renal disease. J. Am. Soc. Nephrol..

[195] Farzi A., Reichmann F., Holzer P. (2015). The homeostatic role of neuropeptide Y in immune function and its impact on mood and behaviour. Acta Physiol. (Oxf.).

[196] Bedoui S., Kromer A., Gebhardt T., Jacobs R., Raber K., Dimitrijevic M., Heine J., von Horsten S. (2008). Neuropeptide Y receptor-specifically modulates human neutrophil function. J. Neuroimmunol..

[197] Dimitrijevic M., Stanojevic S. (2013). The intriguing mission of neuropeptide Y in the immune system. Amino Acids.

[198] Deray G., Carayon A., Maistre G., Benhmida M., Masson F., Barthelemy C., Petitclerc T., Jacobs C. (1992). Endothelin in chronic renal failure. Nephrol. Dial. Transplant..

[199] Zarpelon A.C., Pinto L.G., Cunha T.M., Vieira S.M., Carregaro V., Souza G.R., Silva J.S., Ferreira S.H., Cunha F.Q., Verri W.A. (2012). Endothelin-1 induces neutrophil recruitment in adaptive inflammation via TNFalpha and CXCL1/CXCR2 in mice. Can. J. Physiol. Pharmacol..

[200] Freeman B.D., Machado F.S., Tanowitz H.B., Desruisseaux M.S. (2014). Endothelin-1 and its role in the pathogenesis of infectious diseases. Life Sci..

[201] Zouki C., Baron C., Fournier A., Filep J.G. (1999). Endothelin-1 enhances neutrophil adhesion to human coronary artery endothelial cells: Role of ET(A) receptors and platelet-activating factor. Br. J. Pharmacol..

[202] Sessa W.C., Kaw S., Hecker M., Vane J.R. (1991). The biosynthesis of endothelin-1 by human polymorphonuclear leukocytes. Biochem. Biophys. Res. Commun..

[203] Mencarelli M., Pecorelli A., Carbotti P., Valacchi G., Grasso G., Muscettola M. (2009). Endothelin receptor A expression in human inflammatory cells. Regul. Pept..

[204] Ishida K., Takeshige K., Minakami S. (1990). Endothelin-1 enhances superoxide generation of human neutrophils stimulated by the chemotactic peptide N-formyl-methionyl-leucyl-phenylalanine. Biochem. Biophys. Res. Commun..

[205] Teta D. (2012). Adipokines as uremic toxins. J. Ren. Nutr..

[206] Kopp A., Buechler C., Neumeier M., Weigert J., Aslanidis C., Scholmerich J., Schaffler A. (2009). Innate immunity and adipocyte function: Ligand-specific activation of multiple Toll-like receptors modulates cytokine, adipokine, and chemokine secretion in adipocytes. Obes. (Silver Spring).

[207] Vahdat S. (2018). The complex effects of adipokines in the patients with kidney disease. J. Res. Med. Sci..

[208] Zhu Q., Scherer P.E. (2018). Immunologic and endocrine functions of adipose tissue: Implications for kidney disease. Nat. Rev. Nephrol..

[209] Chan C.C., Damen M., Alarcon P.C., Sanchez-Gurmaches J., Divanovic S. (2019). Inflammation and Immunity: From an Adipocyte’s Perspective. J. Interferon Cytokine Res..

[210] Aminzadeh M.A., Pahl M.V., Barton C.H., Doctor N.S., Vaziri N.D. (2009). Human uraemic plasma stimulates release of leptin and uptake of tumour necrosis factor-alpha in visceral adipocytes. Nephrol. Dial. Transplant..

[211] Alix P.M., Guebre-Egziabher F., Soulage C.O. (2014). Leptin as an uremic toxin: Deleterious role of leptin in chronic kidney disease. Biochimie.

[212] Peng D.Z., Liu X.W., Huang L., Zhu X.F., Zheng Y.Q., Wang L.X. (2014). Relationship between leptin and chronic inflammatory state in uremic patients. Eur. Rev. Med. Pharmacol. Sci..

[213] Cohen G., Raupachova J., Ilic D., Werzowa J., Horl W.H. (2011). Effect of leptin on polymorphonuclear leucocyte functions in healthy subjects and haemodialysis patients. Nephrol. Dial. Transplant..

[214] Curat C.A., Wegner V., Sengenes C., Miranville A., Tonus C., Busse R., Bouloumie A. (2006). Macrophages in human visceral adipose tissue: Increased accumulation in obesity and a source of resistin and visfatin. Diabetologia.

[215] Kunnari A.M., Savolainen E.R., Ukkola O.H., Kesaniemi Y.A., Jokela M.A. (2009). The expression of human resistin in different leucocyte lineages is modulated by LPS and TNFalpha. Regul. Pept..

[216] Miller L., Singbartl K., Chroneos Z.C., Ruiz-Velasco V., Lang C.H., Bonavia A. (2019). Resistin directly inhibits bacterial killing in neutrophils. Intensive Care Med. Exp..

[217] Cohen G., Ilic D., Raupachova J., Hörl W.H. (2008). Resistin inhibits essential functions of polymorphonuclear leukocytes. J. Immunol..

[218] Bostrom E.A., Tarkowski A., Bokarewa M. (2009). Resistin is stored in neutrophil granules being released upon challenge with inflammatory stimuli. Biochim. Biophys. Acta.

[219] Walcher D., Hess K., Berger R., Aleksic M., Heinz P., Bach H., Durst R., Hausauer A., Hombach V., Marx N. (2010). Resistin: A newly identified chemokine for human CD4-positive lymphocytes. Cardiovasc. Res..

[220] Delanghe S., Delanghe J.R., Speeckaert R., Van Biesen W., Speeckaert M.M. (2017). Mechanisms and consequences of carbamoylation. Nat. Rev. Nephrol..

[221] Koeth R.A., Kalantar-Zadeh K., Wang Z., Fu X., Tang W.H., Hazen S.L. (2013). Protein carbamylation predicts mortality in ESRD. J. Am. Soc. Nephrol..

[222] Kraus L.M., Elberger A.J., Handorf C.R., Pabst M.J., Kraus A.P. (1994). Urea-derived cyanate forms epsilon-amino-carbamoyl-lysine (homocitrulline) in leukocyte proteins in patients with end-stage renal disease on peritoneal dialysis. J. Lab. Clin. Med..

[223] Jaisson S., Lorimier S., Ricard-Blum S., Sockalingum G.D., Delevallee-Forte C., Kegelaer G., Manfait M., Garnotel R., Gillery P. (2006). Impact of carbamylation on type I collagen conformational structure and its ability to activate human polymorphonuclear neutrophils. Chem. Biol..

[224] Pavone B., Sirolli V., Giardinelli A., Bucci S., Forli F., Di Cesare M., Sacchetta P., Di Pietro N., Pandolfi A., Urbani A. (2011). Plasma protein carbonylation in chronic uremia. J. Nephrol..

[225] Makita Z., Radoff S., Rayfield E.J., Yang Z., Skolnik E., Delaney V., Friedman E.A., Cerami A., Vlassara H. (1991). Advanced glycosylation end products in patients with diabetic nephropathy. N. Engl. J. Med..

[226] Stinghen A.E., Massy Z.A., Vlassara H., Striker G.E., Boullier A. (2016). Uremic Toxicity of Advanced Glycation End Products in CKD. J. Am. Soc. Nephrol..

[227] Kislinger T., Tanji N., Wendt T., Qu W., Lu Y., Ferran L.J., Taguchi A., Olson K., Bucciarelli L., Goova M. (2001). Receptor for advanced glycation end products mediates inflammation and enhanced expression of tissue factor in vasculature of diabetic apolipoprotein E-null mice. Arterioscler. Thromb. Vasc. Biol..

[228] Cohen G., Rudnicki M., Walter F., Niwa T., Horl W.H. (2001). Glucose-modified proteins modulate essential functions and apoptosis of polymorphonuclear leukocytes. J. Am. Soc. Nephrol..

[229] Glorieux G., Helling R., Henle T., Brunet P., Deppisch R., Lameire N., Vanholder R. (2004). In vitro evidence for immune activating effect of specific AGE structures retained in uremia. Kidney Int..

[230] Kirstein M., Brett J., Radoff S., Ogawa S., Stern D., Vlassara H. (1990). Advanced protein glycosylation induces transendothelial human monocyte chemotaxis and secretion of platelet-derived growth factor: Role in vascular disease of diabetes and aging. Proc. Natl. Acad. Sci. USA.

[231] Rashid G., Korzets Z., Bernheim J. (2006). Advanced glycation end products stimulate tumor necrosis factor-alpha and interleukin-1 beta secretion by peritoneal macrophages in patients on continuous ambulatory peritoneal dialysis. Isr. Med. Assoc. J..

[232] Toure F., Zahm J.M., Garnotel R., Lambert E., Bonnet N., Schmidt A.M., Vitry F., Chanard J., Gillery P., Rieu P. (2008). Receptor for advanced glycation end-products (RAGE) modulates neutrophil adhesion and migration on glycoxidated extracellular matrix. Biochem. J..

[233] D’Agati V., Schmidt A.M. (2010). RAGE and the pathogenesis of chronic kidney disease. Nat. Rev. Nephrol..

[234] Witko-Sarsat V., Gausson V., Descamps-Latscha B. (2003). Are advanced oxidation protein products potential uremic toxins?. Kidney Int. Suppl..

[235] Capeillere-Blandin C., Gausson V., Nguyen A.T., Descamps-Latscha B., Drueke T., Witko-Sarsat V. (2006). Respective role of uraemic toxins and myeloperoxidase in the uraemic state. Nephrol. Dial. Transplant..

[236] Körmöczi G.F., Wolfel U.M., Rosenkranz A.R., Hörl W.H., Oberbauer R., Zlabinger G.J. (2001). Serum proteins modified by neutrophil-derived oxidants as mediators of neutrophil stimulation. J. Immunol..

[237] Jurek A., Turyna B., Kubit P., Klein A. (2008). The ability of HDL to inhibit VCAM-1 expression and oxidized LDL uptake is impaired in renal patients. Clin. Biochem..

[238] Meier P., Golshayan D., Blanc E., Pascual M., Burnier M. (2009). Oxidized LDL modulates apoptosis of regulatory T cells in patients with ESRD. J. Am. Soc. Nephrol..

[239] Donadio C., Tognotti D., Donadio E. (2012). Albumin modification and fragmentation in renal disease. Clin. Chim. Acta.

[240] Mera K., Anraku M., Kitamura K., Nakajou K., Maruyama T., Tomita K., Otagiri M. (2005). Oxidation and carboxy methyl lysine-modification of albumin: Possible involvement in the progression of oxidative stress in hemodialysis patients. Hypertens Res..

[241] Gordon S.M., Hofmann S., Askew D.S., Davidson W.S. (2011). High density lipoprotein: it’s not just about lipid transport anymore. Trends Endocrinol. Metab..

[242] Rosenson R.S., Brewer H.B., Davidson W.S., Fayad Z.A., Fuster V., Goldstein J., Hellerstein M., Jiang X.C., Phillips M.C., Rader D.J. (2012). Cholesterol efflux and atheroprotection: Advancing the concept of reverse cholesterol transport. Circulation.

[243] Navab M., Reddy S.T., Van Lenten B.J., Fogelman A.M. (2011). HDL and cardiovascular disease: Atherogenic and atheroprotective mechanisms. Nat. Rev. Cardiol..

[244] Rye K.A., Barter P.J. (2014). Cardioprotective functions of HDLs. J. Lipid Res..

[245] Creasy K.T., Kane J.P., Malloy M.J. (2018). Emerging roles of HDL in immune function. Curr. Opin. Lipidol..

[246] Norata G.D., Pirillo A., Ammirati E., Catapano A.L. (2012). Emerging role of high density lipoproteins as a player in the immune system. Atherosclerosis.

[247] Schmitz G., Wulf G., Bruning T., Assmann G. (1987). Flow-cytometric determination of high-density-lipoprotein binding sites on human leukocytes. Clin. Chem..

[248] Blackburn W.D., Dohlman J.G., Venkatachalapathi Y.V., Pillion D.J., Koopman W.J., Segrest J.P., Anantharamaiah G.M. (1991). Apolipoprotein A-I decreases neutrophil degranulation and superoxide production. J. Lipid Res..

[249] Murphy A.J., Woollard K.J., Suhartoyo A., Stirzaker R.A., Shaw J., Sviridov D., Chin-Dusting J.P. (2011). Neutrophil activation is attenuated by high-density lipoprotein and apolipoprotein A-I in in vitro and in vivo models of inflammation. Arterioscler. Thromb. Vasc. Biol..

[250] Murphy A.J., Woollard K.J., Hoang A., Mukhamedova N., Stirzaker R.A., McCormick S.P., Remaley A.T., Sviridov D., Chin-Dusting J. (2008). High-density lipoprotein reduces the human monocyte inflammatory response. Arterioscler. Thromb. Vasc. Biol..

[251] Liao X.L., Lou B., Ma J., Wu M.P. (2005). Neutrophils activation can be diminished by apolipoprotein A-I. Life Sci..

[252] Westerterp M., Fotakis P., Ouimet M., Bochem A.E., Zhang H., Molusky M.M., Wang W., Abramowicz S., la Bastide-van Gemert S., Wang N. (2018). Cholesterol Efflux Pathways Suppress Inflammasome Activation, NETosis, and Atherogenesis. Circulation.

[253] Spirig R., Schaub A., Kropf A., Miescher S., Spycher M.O., Rieben R. (2013). Reconstituted high-density lipoprotein modulates activation of human leukocytes. PLoS ONE.

[254] Pirillo A., Catapano A.L., Norata G.D. (2018). Biological consequences of dysfunctional HDL. Curr. Med. Chem..

[255] Marsche G., Saemann M.D., Heinemann A., Holzer M. (2013). Inflammation alters HDL composition and function: Implications for HDL-raising therapies. Pharmacol. Ther..

[256] Saemann M.D., Poglitsch M., Kopecky C., Haidinger M., Horl W.H., Weichhart T. (2010). The versatility of HDL: A crucial anti-inflammatory regulator. Eur. J. Clin. Invest..

[257] Zewinger S., Kleber M.E., Rohrer L., Lehmann M., Triem S., Jennings R.T., Petrakis I., Dressel A., Lepper P.M., Scharnagl H. (2017). Symmetric dimethylarginine, high-density lipoproteins and cardiovascular disease. Eur. Heart J..

[258] Weichhart T., Kopecky C., Kubicek M., Haidinger M., Doller D., Katholnig K., Suarna C., Eller P., Tolle M., Gerner C. (2012). Serum Amyloid A in Uremic HDL Promotes Inflammation. J. Am. Soc. Nephrol..

[259] Florens N., Calzada C., Lyasko E., Juillard L., Soulage C.O. (2016). Modified Lipids and Lipoproteins in Chronic Kidney Disease: A New Class of Uremic Toxins. Toxins (Basel).

[260] Tolle M., Huang T., Schuchardt M., Jankowski V., Prufer N., Jankowski J., Tietge U.J., Zidek W., van der Giet M. (2012). High-density lipoprotein loses its anti-inflammatory capacity by accumulation of pro-inflammatory-serum amyloid A. Cardiovasc. Res..

[261] Raupachova J., Kopecky C., Cohen G. (2019). High-Density Lipoprotein from Chronic Kidney Disease Patients Modulates Polymorphonuclear Leukocytes. Toxins (Basel).

[262] Askari H., Seifi B., Kadkhodaee M., Sanadgol N., Elshiekh M., Ranjbaran M., Ahghari P. (2018). Protective effects of hydrogen sulfide on chronic kidney disease by reducing oxidative stress, inflammation and apoptosis. EXCLI J..

[263] Cao X., Bian J.S. (2016). The Role of Hydrogen Sulfide in Renal System. Front. Pharmacol..

[264] Perna A.F., Luciano M.G., Ingrosso D., Raiola I., Pulzella P., Sepe I., Lanza D., Violetti E., Capasso R., Lombardi C. (2010). Hydrogen Sulfide, the Third Gaseous Signaling Molecule With Cardiovascular Properties, Is Decreased in Hemodialysis Patients. J. Renal Nutr..

[265] Perna A.F., Sepe I., Lanza D., Ingrosso D. (2011). Hydrogen sulfide increases after a single hemodialysis session. Kidney Int..

[266] Vigorito C., Anishchenko E., Mele L., Capolongo G., Trepiccione F., Zacchia M., Lombari P., Capasso R., Ingrosso D., Perna A.F. (2019). Uremic Toxin Lanthionine Interferes with the Transsulfuration Pathway, Angiogenetic Signaling and Increases Intracellular Calcium. Int. J. Mol. Sci..

[267] Lo Faro M.L., Fox B., Whatmore J.L., Winyard P.G., Whiteman M. (2014). Hydrogen sulfide and nitric oxide interactions in inflammation. Nitric Oxide.

[268] Collin M., Anuar F.B., Murch O., Bhatia M., Moore P.K., Thiemermann C. (2005). Inhibition of endogenous hydrogen sulfide formation reduces the organ injury caused by endotoxemia. Br. J. Pharmacol..

[269] Zhang H., Zhi L., Moochhala S., Moore P.K., Bhatia M. (2007). Hydrogen sulfide acts as an inflammatory mediator in cecal ligation and puncture-induced sepsis in mice by upregulating the production of cytokines and chemokines via NF-kappaB. Am. J. Physiol. Lung Cell. Mol. Physiol..

[270] Mok Y.Y., Atan M.S., Yoke Ping C., Zhong Jing W., Bhatia M., Moochhala S., Moore P.K. (2004). Role of hydrogen sulphide in haemorrhagic shock in the rat: Protective effect of inhibitors of hydrogen sulphide biosynthesis. Br. J. Pharmacol..

[271] Rinaldi L., Gobbi G., Pambianco M., Micheloni C., Mirandola P., Vitale M. (2006). Hydrogen sulfide prevents apoptosis of human PMN via inhibition of p38 and caspase 3. Lab. Invest..

[272] Spiller F., Orrico M.I., Nascimento D.C., Czaikoski P.G., Souto F.O., Alves-Filho J.C., Freitas A., Carlos D., Montenegro M.F., Neto A.F. (2010). Hydrogen sulfide improves neutrophil migration and survival in sepsis via K+ATP channel activation. Am. J. Respir. Crit. Care Med..

[273] Wallace J.L., Ferraz J.G., Muscara M.N. (2012). Hydrogen sulfide: An endogenous mediator of resolution of inflammation and injury. Antioxid. Redox Signal..

[274] Zanardo R.C., Brancaleone V., Distrutti E., Fiorucci S., Cirino G., Wallace J.L. (2006). Hydrogen sulfide is an endogenous modulator of leukocyte-mediated inflammation. Faseb. J..

[275] Faller S., Hausler F., Goeft A., von Itter M.A., Gyllenram V., Hoetzel A., Spassov S.G. (2018). Hydrogen sulfide limits neutrophil transmigration, inflamm.ation, and oxidative burst in lipopolysaccharide-induced acute lung injury. Sci. Rep..

